# Kicking against the PRCs – A Domesticated Transposase Antagonises Silencing Mediated by Polycomb Group Proteins and Is an Accessory Component of Polycomb Repressive Complex 2

**DOI:** 10.1371/journal.pgen.1005660

**Published:** 2015-12-07

**Authors:** Shih Chieh Liang, Ben Hartwig, Pumi Perera, Santiago Mora-García, Erica de Leau, Harry Thornton, Flavia Lima de Alves, Juri Rapsilber, Suxin Yang, Geo Velikkakam James, Korbinian Schneeberger, E. Jean Finnegan, Franziska Turck, Justin Goodrich

**Affiliations:** 1 Institute of Molecular Plant Science, University of Edinburgh, Edinburgh, United Kingdom; 2 Department of Plant Developmental Biology, Max Planck Institute for Plant Breeding Research, Köln, Germany; 3 Wellcome Trust Centre for Cell Biology, University of Edinburgh, Edinburgh, United Kingdom; 4 CSIRO Plant Industry, Canberra, Australia; University of Utah School of Medicine, UNITED STATES

## Abstract

The Polycomb group (PcG) and trithorax group (trxG) genes play crucial roles in development by regulating expression of homeotic and other genes controlling cell fate. Both groups catalyse modifications of chromatin, particularly histone methylation, leading to epigenetic changes that affect gene activity. The trxG antagonizes the function of PcG genes by activating PcG target genes, and consequently trxG mutants suppress PcG mutant phenotypes. We previously identified the *ANTAGONIST OF LIKE HETEROCHROMATIN PROTEIN1* (*ALP1*) gene as a genetic suppressor of mutants in the Arabidopsis PcG gene *LIKE HETEROCHROMATIN PROTEIN1* (*LHP1*). Here, we show that *ALP1* interacts genetically with several other PcG and trxG components and that it antagonizes PcG silencing. Transcriptional profiling reveals that when PcG activity is compromised numerous target genes are hyper-activated in seedlings and that in most cases this requires *ALP1*. Furthermore, when PcG activity is present *ALP1* is needed for full activation of several floral homeotic genes that are repressed by the PcG. Strikingly, *ALP1* does not encode a known chromatin protein but rather a protein related to *PIF/Harbinger* class transposases. Phylogenetic analysis indicates that ALP1 is broadly conserved in land plants and likely lost transposase activity and acquired a novel function during angiosperm evolution. Consistent with this, immunoprecipitation and mass spectrometry (IP-MS) show that ALP1 associates, *in vivo*, with core components of POLYCOMB REPRESSIVE COMPLEX 2 (PRC2), a widely conserved PcG protein complex which functions as a H3K27me3 histone methyltransferase. Furthermore, in reciprocal pulldowns using the histone methyltransferase CURLY LEAF (CLF), we identify not only ALP1 and the core PRC2 components but also plant-specific accessory components including EMBRYONIC FLOWER 1 (EMF1), a transcriptional repressor previously associated with PRC1-like complexes. Taken together our data suggest that ALP1 inhibits PcG silencing by blocking the interaction of the core PRC2 with accessory components that promote its HMTase activity or its role in inhibiting transcription. ALP1 is the first example of a domesticated transposase acquiring a novel function as a PcG component. The antagonistic interaction of a modified transposase with the PcG machinery is novel and may have arisen as a means for the cognate transposon to evade host surveillance or for the host to exploit features of the transposition machinery beneficial for epigenetic regulation of gene activity.

## Author Summary

Transposons are parasitic genetic elements that proliferate within their hosts’ genomes. Because rampant transposition is usually deleterious, hosts have evolved ways to inhibit the activity of transposons. In plants, this genome defence is provided by the Polycomb group (PcG) proteins and/or the DNA methylation machinery, which repress the transcription of transposase genes. We identified the *Arabidopsis ALP1* gene through its role in opposing gene silencing mediated by PcG genes. *ALP1* is an ancient gene in land plants and has evolved from a domesticated transposase. Unexpectedly, we find that the ALP1 protein is present in a conserved complex of PcG proteins that inhibit transcription by methylating the histone proteins that package DNA. ALP1 likely inhibits the activity of this PcG complex by blocking its interaction with accessory proteins that stimulate its activity. We suggest that the inhibition of the PcG by a transposase may originally have evolved as a means for transposons to evade surveillance by their hosts, and that subsequently hosts may have exploited this as a means to regulate PcG activity. Our work illustrates how transposons can be friend or fiend, and raises the question of whether other transposases will also be found to inhibit their host’s regulatory machinery.

## Introduction

The Polycomb group (PcG) genes are widely conserved in plants and animals and mediate an epigenetic system for repressing transcription of developmental patterning and other target genes. They were originally identified from genetic studies in *Drosophila* [2] by virtue of their shared role in repressing homeotic genes and subsequently discovered in other organisms, often through a similar role in controlling developmental patterning and mediating epigenetic transcriptional silencing. Although stable, PcG-mediated silencing can be reversed, most commonly between generations during germline or early embryo development but also during somatic development [3]. Two outstanding questions are how does the PcG mediate transcriptional silencing and how is this overturned?

PcG mediated gene silencing is strongly associated with histone methylation, specifically trimethylation of lysine 27 on the amino tail of histone H3 (H3K27me3) [4]. This modification is catalysed by Polycomb Repressive Complex 2 (PRC2), that comprises four widely conserved PcG proteins, which in *Drosophila* are Enhancer of zeste [E(z)], Extra sex combs (Esc), Suppressor of zeste 12 [Su(z)12] and Nurf55 [5,6]. In *Arabidopsis* the different members are represented by small gene families: for example the catalytic subunit E(z) is encoded by the three genes *MEDEA* (*MEA*), *CURLY LEAF* (*CLF*) and *SWINGER* (*SWN*); similarly, the Su(z)12 subunit is encoded by the three genes *EMBRYONIC FLOWER2* (*EMF2*), *VERNALIZATION2* (*VRN2*) and *FERTILIZATION INDEPENDENT SEED DEVELOPMENT2* (*FIS2*). *MEA* and *FIS2* act specifically in seed, whereas *CLF* and *SWN* show overlapping and partially redundant roles in the plant body as do *EMF2* and *VRN2* [7,8]. Although best known as a histone mark “writer”, it has recently emerged that the PRC2 has other activities towards chromatin including as a “reader” of marks. Thus the Esc component can specifically bind H3K27me3 and when bound it stimulates the histone methyltransferase (HMTase) activity of PRC2 [9]. By contrast, the Su(z)12 component can bind the antagonistic marks H3K4me3 and H3K36me3 that are associated with active genes, and this can result in downregulation of the HMTase activity of PRC2 [10]. This interplay between reading and writing activities within a single complex likely helps reinforce alternative stable chromatin states marked by active or repressive marks. Whilst the four core components of PRC2 are very widely conserved throughout metazoans and land plants, various accessory components have been identified that are usually more restricted. For example, in animals the DNA binding protein AEBP2 (JING in *Drosophila*) is a component of PRC2 and may have a role in recruiting PRC2 to chromatin marked with mono-ubiquitinated histone H2A (H2Aub) and stimulating HMTase activity [11]. In *Arabidopsis*, the PHD domain containing protein VERNALIZATION INSENSITIVE 3 (VIN3), and three related proteins VIN3-like 1–3 (VIL1-3, also called VRN5, VEL1 and VEL2 respectively) can associate with PRC2 and are thought to upregulate HMTase activity [12].

Although H3K27me3 methylation is necessary for silencing by the PcG, it does not directly inhibit transcription and there are several examples where H3K27me3 decorated targets are activated without removal of this mark [13–15]. This suggests that other PcG proteins have more direct roles in transcriptional silencing. Indeed, a second PcG protein complex, Polycomb Repressive Complex 1 (PRC1) has been shown to have several activities on chromatin that inhibit transcription. Firstly, purified PRC1 has several non-covalent activities towards chromatin templates *in vitro* including inhibiting chromatin remodeling, promoting chromatin compaction and also inhibiting transcription [16–19]; the role of PRC1 in chromatin compaction has also been demonstrated *in vivo* [13]. The canonical PRC1 contains four proteins, in *Drosophila*: Polycomb (Pc), Posterior Sex Comb (Psc), Polyhomeotic (Ph) and Sex Combs Extra (Sce) [19]. A poorly conserved, C-terminal region of Psc is sufficient for all of these non-covalent activities of the PRC1 in silencing, at least *in vitro*. Secondly, two PRC1 components—Sce and the N-terminal portion of Psc in *Drosophila*–have RING finger domains with E3 ubiquitin ligase activity and promote H2Aub ubiquitination most notably when in a variant PRC1 complex termed dRAF [20]. The H2Aub modification may inhibit transcription by blocking the recruitment of factors needed for RNA PolII-dependent transcriptional elongation at target gene promoters [21,22]. Genetic analysis in which the E3 ligase activity of Sce orthologues was specifically mutated in mouse embryonic stem cells abolished H2Aub *in vivo* and caused derepression of many PcG targets, confirming that H2Aub is relevant for PcG silencing [23]. However, chromatin compaction and partial repression was maintained at *Hox* gene targets, suggesting that the two roles of PRC1 in silencing are partially separable. Furthermore, similar experiments in Drosophila have shown that whilst H2Aub is required for viability, it is dispensable for silencing of canonical PcG targets [24]. The PRC1 members are less well conserved in plants than the PRC2, however similar proteins and activities have been found in *Arabidopsis* [25]. For example, LIKE HETEROCHROMATIN PROTEIN 1 (LHP1) is equivalent but not homologous to Pc, and like Pc it can bind H3K27me3 via a chromodomain [26]. The plant specific PcG protein EMBRYONIC FLOWER1 (EMF1) is unrelated to Psc but has similar architectural features to the Psc C-terminal region and likely has a similar role in silencing: like Psc it has been shown to inhibit chromatin remodeling and transcription *in vitro* and it is required for the silencing of many PcG targets *in vivo* [27,28]. The *Arabidopsis* AtBMI1 and AtRING1 proteins, orthologues of Psc and Sce, respectively, mediate H2Aub and are needed for the silencing of a subset of PcG target genes [29–31]. Some PcG targets that are heavily H3K27me3 methylated such as *FUSCA 3* (*FUS3*) are strongly dependent on *AtBMI1/AtRING1* but not *EMF1* for transcriptional repression, whereas others such as *AGAMOUS* (*AG*) require *EMF1* but not *AtBMI1/AtRING1* suggesting two partially independent pathways by which H3K27me3 methylated genes are silenced [25]. Whether the plant PRC1 members also co-associate in PRC1-like complexes *in vivo* is unclear. EMF1 and LHP1 co-immunoprecipitate from plant extracts [32], and both LHP1 and EMF1 interact with AtRING1 and AtBMI1 proteins in *in vitro* pull down assays [29]. Additionally, EMF1 is required for AtRING1/AtBMI1 mediated H2Aub *in vivo* [29]. Together, these observations suggest that a PRC1 like complex containing EMF1/LHP1/AtRING1/AtBMI1 may occur in plants. However, LHP1 was also found to co-purify with MSI1 and other PRC2 components when MSI1 was immunoprecipitated from cross-linked protein extracts [33] and EMF1 also interacts with MSI1 in *in vitro* pull down assays [28]. Thus some PRC1 members may also have roles in PRC2 complexes in plants, or PRC1 and PRC2 complexes may be less distinct.

A second group of genes, termed the trithorax group (trxG), acts as antagonists of PcG silencing and promotes the stable activation of PcG targets. A defining genetic property of trxG mutants is that they suppress PcG mutant phenotypes, as they are required for the transcriptional activation of PcG targets [34]. Although the trxG has been less extensively characterized in plants than the PcG, several members have been identified from forward and reverse genetic screens and in several cases have biochemical activities towards chromatin that are opposite to those of the PcG. For example, *RELATIVE OF EARLY FLOWERING 6* (*REF6*) encodes a jumonji domain protein which demethylates the H3K27me3 and H3K27me2 modifications catalyzed by the PRC2 and genetically acts a suppressor of mutants in *CLF*, encoding the catalytic subunit of PRC2 [35]. *ARABIDOPSIS TRITHORAX LIKE 1* (*ATX1*) and *EARLY FLOWERING IN SHORT DAYS* (*EFS*) encode HMTases that deposit H3K4me3 and H3K36me3, respectively, two marks associated with transcriptional activity that are known to inhibit the H3K27me3 HMTase activity of the PRC2 [36,37]. The plant-specific *ULTRAPETALA1* (*ULT1*) gene also antagonizes *CLF* genetically and can activate *CLF* target genes when overexpressed. The biochemical function of ULT1 is unclear but it has been found to interact with ATX1 and may therefore have a role in promoting H3K4 methylation [38]. The related *BRAHMA* and *SPLAYED* genes act as genetic suppressors of *clf* mutants and are required to overcome PcG repression of floral homeotic genes during flower development. They encode SWI2/SNF2 chromatin remodelers i.e. an activity opposite to that of EMF1, which inhibits chromatin remodeling [39].

To further identify genes antagonizing PcG silencing we previously carried out a genetic screen for suppressors of the lhp1 mutant phenotype in *Arabidopsis* and so identified *ANTAGONIST OF LIKE HETEROCHROMATIN PROTEIN1* (*ALP1*) [1]. Here we perform a detailed genetic, molecular and proteomic characterization. We show genetically that *ALP1* interacts with various PcG and trxG members and through transcriptional profiling that it is required for activity of the majority of *CLF* target genes. The relationship of *ALP1* with chromatin was previously uncertain, as it was found to encode a domesticated transposase. Using proteomics we show that CLF is associated not just with the core PRC2 members but also with ALP1, LHP1 and EMF1 *in vivo*. By contrast, ALP1 associates with the core components of the CLF and SWN containing PRC2 complexes *in vivo* but not with EMF1 and LHP1. This suggests that ALP1 may antagonize PRC2 silencing by inhibiting the interaction with EMF1 and or LHP1. The association of a domesticated transposase with the PcG machinery is novel and raises the question of whether transposases may more generally have evolved roles in inhibiting epigenetic machinery as a way to evade host surveillance and/or the hosts exploited this as a means to regulate the PcG.

## Results

### *alp1* mutants suppress defects in several PRC1 and PRC2 components

**Fig 1:**
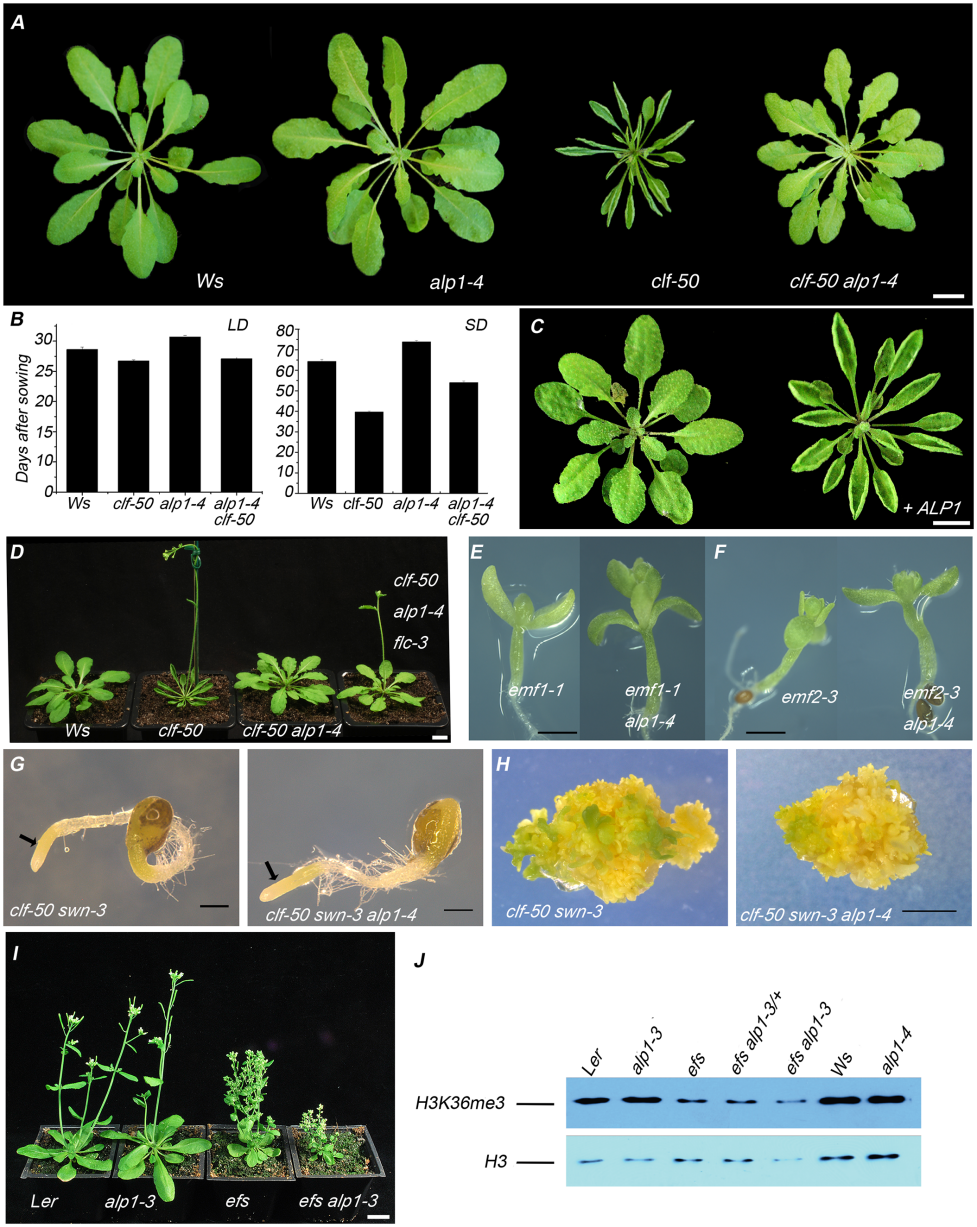
Genetic interaction of *ALP1* with PcG. (A) *alp1-4* suppresses the clf phenotype. Short day grown plants at six weeks old. (B) Effect of *alp1-4* on flowering time. The *alp1-4* mutation partially suppresses the early flowering of *clf-50* in short days. (C) The *gALP1* transgene complements the *alp1-4* mutation. The two plants are *clf-50 alp1-4* siblings from a T3 family segregating the transgene. The plant with the g*ALP1* transgene has a restored clf phenotype i.e. the suppression caused by the *alp1-4* mutation has been complemented. (D) *FLC* activity is not required for the suppression of the clf phenotype by *alp1-4*. Thus *clf-50 alp1-4 flc-3* triple mutants have a similar supressed clf phenotype as *clf-50 alp1-4* double mutants but are slightly earlier flowering due to reduced *FLC* activity. (E-F) Double mutants of *alp1-4* with either *emf1-1* or *emf2-3* fail to suppress the characteristic emf phenotype of minute plants which lack rosette leaves. (G-H) The *alp1-4* mutation does not suppress the clf swn phenotype including the “pickle” root phenotype (arrowed in G) or the proliferation of callus-like tissue (H) when grown in tissue culture. (I) The *alp1-3* mutation enhances the short branched phenotype of *efs* mutants. (J) Western blot analysis of total histone protein extracts analysed with antibodies to H3K36me3. The blot was stripped and reanalysed with an antibody to histone H3 to check the total loading of histone proteins in each lane (lower panel). The level of H3K36me3 is decreased in *efs* mutants relative to the wild type progenitor. However, neither *alp1-4* nor *alp1-3* mutations reduce H3K36me3 levels relative to their wild-type progenitors. In addition, removing *ALP1* activity in the *efs* mutant background does not cause any further decrease in H3K36me3 (the apparent slight decrease reflects differences in loading as revealed in the lower panel). Scale bars are 1 cm in A, C, D, I and in E, F, G and H are 1mm.

*ALP1* was first identified in a genetic screen for suppressors of the *Arabidopsis* PcG mutant *lhp1* [1]. Independently, we identified *ALP1* in a second genetic screen [described previously in 40] for suppressors of *clf* mutants. *CLF* encodes the catalytic component of the PRC2 and acts largely redundantly with the closely related *SWN* gene [15,41]. Similar to the results for *alp1 lhp1* plants, *alp1* partially suppressed the clf mutant phenotype. The suppression was clearest in short day conditions, where the clf phenotype is milder than in long days; the *alp1-4 clf-50* double mutants closely resembled wild type plants with larger, less curled leaves than *clf-50* mutants (Fig 1A) and were intermediate in flowering time between *clf-50* and wild-type in SD (Fig 1B). Molecular analysis indicated that *alp1-4* was caused by a T-DNA insertion in the third exon of *ALP1* (S1A Fig). To confirm that the suppression of the clf phenotype was caused by *alp1-4* rather than any other mutation in the background, we introduced a transgene (*gALP1*) containing 2.9 kb of genomic DNA spanning the *ALP1* locus, into the *clf-50 alp1-4* background; this complemented the *alp1-4* mutation i.e. *clf-50 alp1-4 gALP1* plants, unlike *clf-50 alp1-4*, showed a severe clf mutant phenotype (Fig 1C). In addition, we found that an independent *alp1* mutation isolated in a different genetic background (*alp1-3* in L*er*) also gave a partial suppression of the clf phenotype (S1B Fig).

Our previous analysis showed that many mutants which suppress the clf phenotype are very late flowering in both *clf* and wild-type backgrounds; their suppression is caused by high levels of the *FLOWERING LOCUS (FLC)* gene which represses *FLOWERING LOCUS T (FT)* and other key targets of CLF [40]. To test whether *ALP1* also affected flowering, we characterised *alp1* mutants in a wild-type (*CLF* ^*+*^) background. The *alp1-4* single mutant had normal flowers and showed no aberrant morphological phenotype (Fig 1A); although leaves of *alp1-4* occasionally showed weak downward curling we were not able to reliably distinguish mutants from wild-type siblings in segregating populations. The *alp1-4* mutants had normal flowering time in long days, and in short days were on average slightly late flowering (Fig 1B), but there was considerable overlap in flowering time between mutant and wild type. Importantly, the suppression of *clf* by *alp1* was not dependent on *FLC* activity, as although *clf-50 alp1-4 flc-3* triple mutants flowered earlier than *clf alp1-4* they nonetheless retained a suppressed clf phenotype (Fig 1D).

To test whether *ALP1* interacts more generally with the plant PcG, we combined *alp1* with other PcG mutants. The *emf2* and *emf1* mutants have a more severe phenotype than *clf* or *lhp1*, but regulate similar targets in the flowering pathway [33,42]. However, *alp1* mutations did not suppress either *emf2* or *emf1* mutants (Fig 1E and 1F). In addition, *alp1* did not suppress the severe *clf swn* double mutant, in which PRC2 activity and H3K27me3 methylation is eliminated from plants (Fig 1G and 1H) [43]. The *MEA* gene is closely related to *CLF* and *SWN* and is specifically expressed in the central cell of the female gametophyte and the descendant endosperm; in *mea/+* heterozygotes about 50% of seeds abort (those inheriting the defective allele maternally) [8]. To test whether *alp1* can suppress *mea* mutations, we made *alp1-4 mea-emb173/+* plants. Similar to *mea-emb173/+* plants, about 50% of seed on these plants were shrivelled (1778:1522 shrivelled:non-shrivelled, 54%, see also S2A Fig). Thus the *alp1* mutation did not obviously suppress the seed abortion phenotype conferred by *mea*, although we cannot rule out subtle effects on the mea phenotype.

Since the genetic suppression of PcG mutants is a defining property of trxG genes, *ALP1* may represent a novel plant trxG member: we therefore tested whether *alp1* enhanced plant trxG mutants in double mutant combinations. Double mutants of *alp1* with mutants in the *ATX1* gene did not enhance the atx1 phenotype (S2B and S2C Fig). By contrast, *alp1* enhanced the floral phenotype of mutants in both *ULT1* and the closely related *ULT2* gene, so that the double mutants had more floral organs than *ult1* or *ult2* single mutants (S3A, S3B and S4 Figs). Since *alp1* single mutants have normal floral organ number, *alp1* interacts synergistically with *ult1* and *ult2*. The increased floral organ number in *ult* mutants is thought to be due to impaired activation of the floral homeotic gene *AG*, which results in prolonged activity of the *WUSCHEL* (*WUS*) gene promoting stem cell activity and increasing meristem size and organ number [44,45]. *ALP1* antagonises *CLF*, which is a repressor of *AG* expression. The enhancement of *ult* mutations by *alp1* might therefore occur if *ALP1* and *ULT* act in parallel in activating *AG*. In addition, *alp1-3* strongly enhanced the dwarf, branched phenotype of mutants in the *EFS* gene encoding an H3K36me3 histone methyltransferase, although *alp1* single mutants had normal height and branching (Fig 1I): for example, whereas Ler and *alp1-3* had a similar mean total branch number (4.0 vs 3.18, P>0.05), *alp1-3 efs* had significantly more branches than *efs* plants (34.4 vs 24.4, P<0.05). In progeny of an *efs alp1-3/+* individual we observed 17 plants with the enhanced phenotype and 39 with the less severe phenotype; genotyping showing that 16 of the 17 plants with the severe phenotype were *alp1-3* homozygotes whereas all 39 of the less severe were *ALP1+* homozygotes or heterozygotes, consistent with *alp1-3* causing the enhancement. Finally, we made double mutants in Col-0 background between *alp1-4* and an independent *efs* allele and observed a similar enhancement (S3C Fig). Together, these data indicate a non-additive genetic interaction, consistent with *ALP1* acting in parallel with *EFS* on common targets. In western blot analysis of total histone extracts we found that although the *efs* mutation reduced global H3K36me3 levels as previously reported [37], *alp1* mutations did not have any effect (Fig 1J), again suggesting the two genes act independently. Collectively, the fact that the effects of *alp1* mutation were most apparent in specific PcG and trxG backgrounds suggested that *ALP1* has an activity towards chromatin, and that genetically it behaves as a trxG member.

### *ALP1* is ancient and conserved throughout land plants

**Fig 2:**
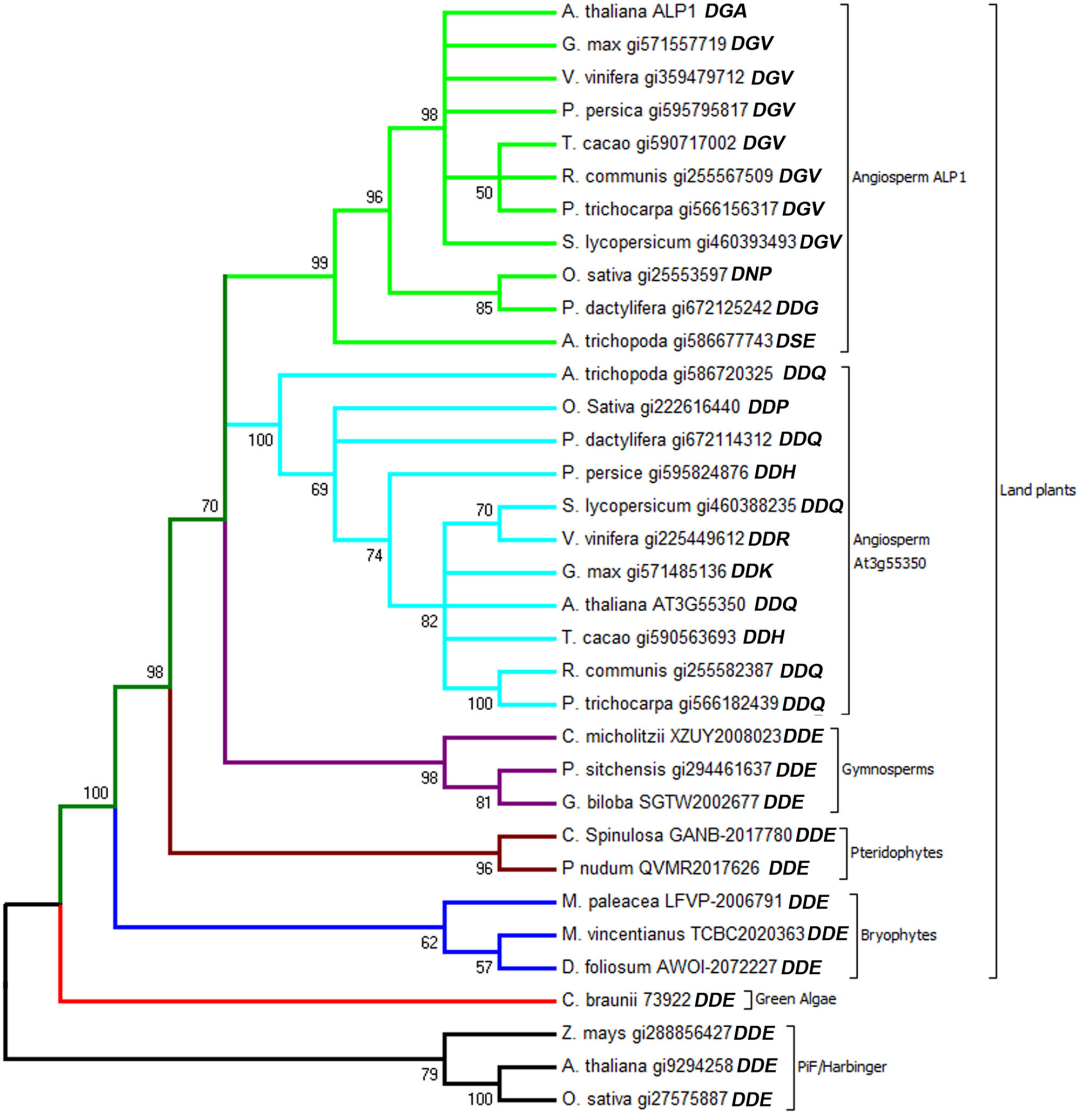
Phylogenetic analysis of ALP1 sequences from land plants and green algae. Molecular phylogenetic analysis by maximum likelihood (ML) method implemented in MEGA6 [74]. The bootstrap consensus tree inferred from 200 replicates is taken to represent the evolutionary history of the taxa analyzed. Branches corresponding to partitions reproduced in less than 50% bootstrap replicates are collapsed. The composition of the DDE catalytic triad is indicated on the tips of the branches. The tree is unrooted. The species indicated are *Arabidopsis thaliana*, *Glycine max* (soybean), *Vitis vinifera* (grape), *Prunus persica* (peach), *Theobroma cacao* (cacao), *Ricinus communis* (castor bean), *Populus trichocarpa* (poplar), *Solanum lycopersicon* (tomato), *Oryza sativa* (rice), *Phoenix dactylifera* (date palm), *Amborella trichopoda*, *Cycas micholitzii*, *Picea sitchensis*, *Ginkgo biloba*, *Cyathea spinulosa*, *Psilotum nudum*, *Marchantia paleacea*, *Diphyscium foliosum*, *Nothoceros vincentianus*, *Chara braunii* and *Zea mays* (maize). PIF/Harbinger transposase branches are coloured in black, those of green algae in red, bryophytes in blue, pteridophytes in orange, gymnosperms in magenta, the angiosperm ALP1 clade in green, the angiosperm At3g55350 clade in light blue. Genbank accession numbers are prefixed GI, others are accession numbers for sequence retrieved from the 1000 plant genomes website (www.onekp.com) with the exception of the *Chara braunii* sequence which is given the contig number in the transcriptome assembly. The analysis involved 34 amino acid sequences. All positions with less than 95% site coverage were eliminated. That is, fewer than 5% alignment gaps, missing data, and ambiguous residues were allowed at any position. There were a total of 323 positions in the final dataset.

We previously reported that *ALP1* is plant-specific, conserved in higher plants (eudicots) and encodes a 396 amino acid protein with similarity to a tranposase encoded by the *PIF/Harbinger* superfamily of transposons in plants and animals [1]. *PIF/Harbinger* transposons encode two proteins, one with DNA binding activity and the other a transposase with DNA endonuclease activity [46–48], whereas in most other DNA transposon families both activities are combined in a single protein. ALP1 is related to the transposase component, but is unlikely to retain activity as it has non-conservative substitutions (DGA in place of DDE) for two of the three acidic residues that comprise a highly conserved catalytic triad involved in metal ion co-ordination at the active site of transposases and other endonucleases [1, see also Fig 2 and S5 Fig]. To test whether *ALP1* is conserved outside eudicots we queried EST sequence databases from monocots, basal angiosperms, gymnosperms, ferns, bryophtyes and green algae. We identified proteins similar to ALP1 in all major land plant groups. All the ALP1-related proteins retrieved ALP1 as the best hit in reciprocal BLAST searches against the *Arabidopsis* genome, suggesting they were ALP1 homologues. Alignments between land plant ALP1 proteins revealed blocks of conserved sequence in similar positions to the regions previously found to be conserved between PIF/Harbinger transposases (S5 Fig). We previously identified a potential DNA binding motif within a conserved region of ALP1 [residues 108–138, see 1]. Structural prediction, using the PHYRE program [49], suggests that an overlapping region (residues 96–142) has a similar structure to the homeodomain of Centromere Protein B, a DNA binding protein also related to transposases. However the sequence similarity between the two proteins in this region is very low (17% identity over 47 amino acids). We were unable to identify any other motifs associated with chromatin modification or transcriptional regulation.

To further analyse the relationship between ALP1-like proteins and *PIF/Harbinger* transposases we constructed phylogenetic trees based on the aligned protein sequences. This revealed that the land plant ALP1 proteins form a strongly supported group (bootstrap value 100) distinct from that of *PIF/Harbinger* transposases (Fig 2). Several other observations further suggested that the ALP1 homologues are unlikely to be components of functional transposons: firstly, they were single copy in virtually all genomes queried, unlike autonomous *PIF/Harbinger* transposons which typically occur at much higher copy number in plants [50]; secondly, in cases where flanking genomic sequences were available we found that the *ALP1* genes lacked the neighbouring gene encoding a DNA binding protein that is characteristic of *PIF/Harbinger* transposons; finally, comparison of the genomic sequences flanking *ALP1* between *Arabidopsis thaliana*, *Arabidopsis lyrata* and *Populus trichocarpa* (poplar, phylogenetically close to Brassicaceae) reveals that *ALP1* is in a syntenic region in all three genomes (S6A Fig) and is therefore immobile. Collectively, these data suggest that *ALP1* arose by domestication of a *PIF/Harbinger* type transposase gene and was present in the common ancestor of all land plants. We further identified a sequence from the green algae *Chara braunii* with similarity to ALP1. This was not well resolved in our tree, but occupies an intermediate position between ALP1 and PIF/Harbinger, and may share a more recent common origin with ALP1 than the transposases.

Within angiosperms, ALP1 is represented by an ALP1 clade and a sister clade that includes AT3G55350, the *Arabidopsis* protein most similar to ALP1 (Fig 2). The genes in these two clades contain a single intron which is located at an identical position near the 5’-end of the coding sequence, further supporting that they have a recent common origin (S6B Fig). In both clades, one or two of the three residues in the DDE catalytic triad that is conserved in functional transposases have been mutated (Fig 2). By contrast, in all the land plant groups basal to the angiosperms the DDE triad is conserved (Fig 2). This suggests that during angiosperm evolution ALP1 lost endonuclease activity and acquired a novel function.

The similarity between *ALP1* and *At3g55350* raised the possibility that the two genes act redundantly. To test this we made double mutants between *alp1-1* and a T-DNA insertion allele of *At3g55350* (Salk 122829), however we did not observe any obvious enhancement of the alp1 mutant phenotype (S1 Fig).

### ALP1 activates PcG targets in *clf* mutant backgrounds

**Fig 3:**
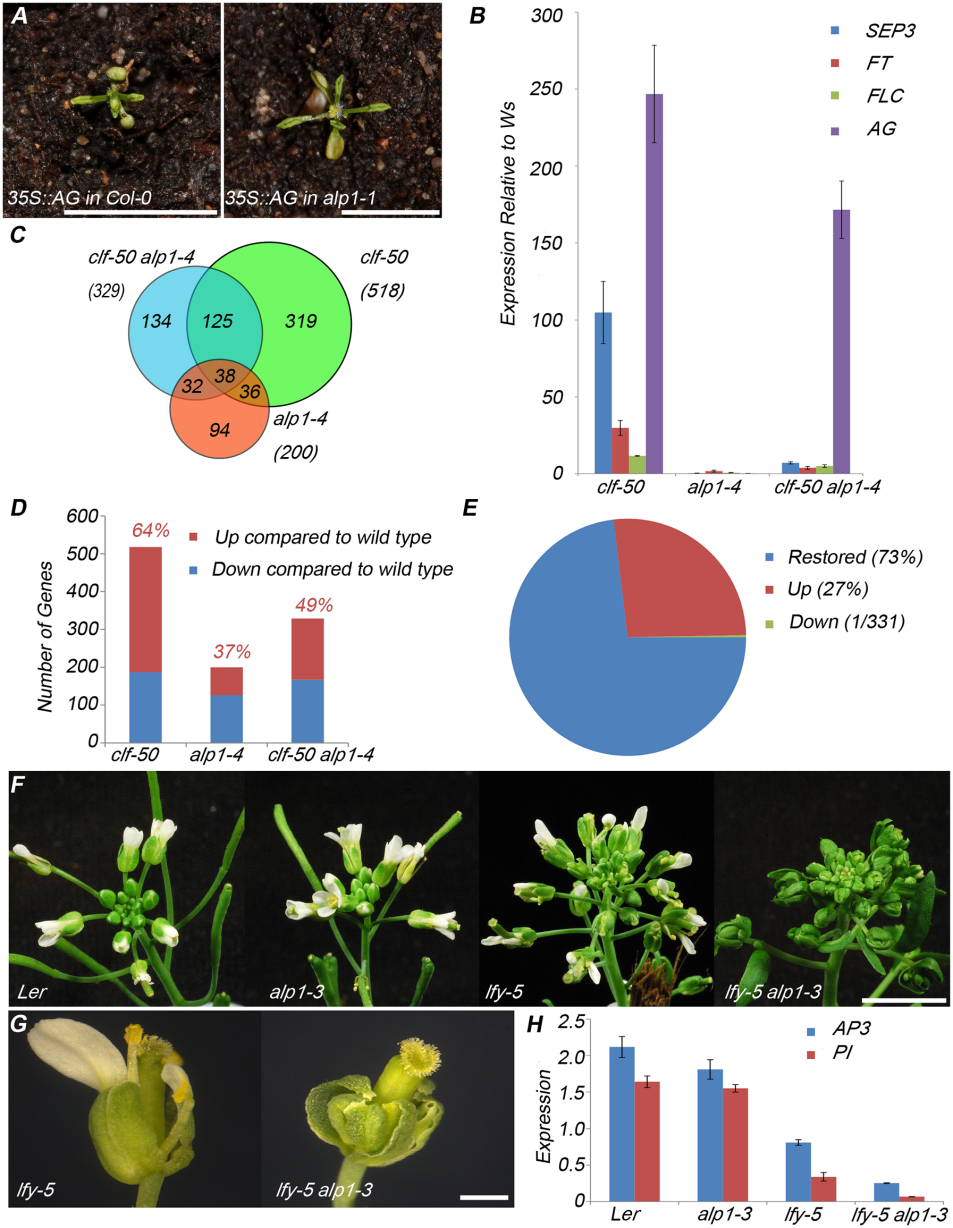
*ALP1* is required to activate PcG target gene expression. (A) T1 plants transformed with *35S*::*AG* transgene. The *alp1-1* mutation did not suppress the characteristic phenotype of small, early flowering plants with narrow, curled leaves. (B) Real time RT-PCR analysis of *SEP3*, *FT*, *FLC* and *AG* expression in seedlings of 12 day old short day grown plants. Relative expression was first normalised relative to the *EiF4A* reference gene and then calculated relative to the wild type value. Error bars indicate the standard error of the mean of three biological replicates. All four genes are upregulated in *clf-50* but show reduced expression in *clf-50 alp1-4* double mutants. One way ANOVA tests indicate that the differences are significant (p<0.05) between *clf-50* and *clf-50 alp1-4* for *SEP3*, *FT* and *FLC* but not *AG*. (C) Venn diagram comparing the number of genes mis-regulated relative to wild type (Ws) in 12 day old seedlings. Misregulated genes showed Log_2_(FoldChange)>2 and False Discovery Rate <0.05. (D) Bar charts comparing the number of genes downregulated (blue) and up-regulated (red) relative to wild-type. Numbers above the bars indicate the proportion of up-regulated genes. (E) Pie chart showing that the bulk of genes mis-expressed in *clf-50* relative to wild-type are no longer mis-expressed (restored) in *clf-50 alp1-4* relative to wild-type. (F-G) Inflorescences (F) and flowers (G) illustrating the enhancement of the weak *lfy-5* phenotype by *alp1-3*. In *lfy-5* flowers, fewer petals and stamens are produced than in wild-type whereas *lfy-5 alp1-3* flowers from similar position on the inflorescence had much more severe phenotype with petals and stamens usually lacking and replaced with sepals and carpels, respectively (G). (H) Real time RT-PCR analysis of *AP3* and *PI* expression in inflorescences shows reduced expression of both genes in *lfy-5* compared to wild type and a more severe reduction in *lfy-5 alp1-3* consistent with the enhanced phenotype. Expression is normalised relative to the reference gene *EIF4A*. Error bars indicate standard error of mean of three biological replicates. Scale bars are 5mm in A and F, 500μm in G.

The suppression of PcG mutant phenotypes by *alp1* suggested that *ALP1* is required for the activity of PcG targets. One possibility is that *ALP1* acts downstream of targets, for example to mediate their activity in conferring leaf curling. To test whether *ALP1* is needed for downstream function of *AG*, a key target of *CLF* and *LHP1*, we introduced the *35S*::*AG* transgene into wild-type and *alp1* mutant backgrounds. The *35S*::*AG* transgene confers a strong leaf curling phenotype, similar to that of *clf* mutants, due to *AG* mis-expression in leaves [51]. We observed a similar leaf curling phenotype in both wild-type and *alp1* backgrounds (Fig 3A) suggesting that *ALP1* was not required downstream of *AG* for its activity. To test whether *ALP1* is required upstream of PcG targets for their transcriptional activation we first quantified gene expression of the key *CLF* targets *AG*, *SEPALLATA3 (SEP3)*, *FT* and *FLC* by real time RT-PCR (Fig 3B). As previously described, expression of all four genes was strongly increased in *clf-50* relative to wild-type seedlings. All four genes were less strongly mis-expressed in *clf-50 alp1-4* than in *clf-50* consistent with ALP1 acting as a transcriptional activator of PcG targets. To test more globally whether *ALP1* was needed for PcG target activity, we compared the transcriptomes of wild-type (Ws ecotype), *clf-50*, *alp1-4* and *clf-50 alp1-4* plants seedlings. In comparison to wild-type, more genes were mis-regulated in *clf-50* than in *clf-50 alp1-4* or *alp1-4* mutants, consistent with the more severe phenotype of *clf-50* (Fig 3C). More genes were upregulated than downregulated in *clf-50*, consistent with the role of *CLF* as a repressor, and the up-regulated genes included known *CLF* targets (Fig 3D, see also S1 Table). Strikingly, of the 331 genes up-regulated in *clf-50*, the majority (73%) were no longer up-regulated in *clf-50 alp1-4* double mutants (Fig 3E). Therefore, *ALP1* is generally required for the activation of PcG targets when *CLF* activity is lacking.

In comparisons of *alp1-4* with wild-type, more genes were downregulated than up (Fig 3D), suggesting that *ALP1* may also have a role as an activator in *CLF* ^+^ backgrounds. Furthermore, of the 126 genes that are downregulated in *alp1-4*, 57 are enriched for H3K27me3 (based on [52]), a much higher fraction than the genome average (p<2.6 x E-16, hypergeometric test), consistent with a role for *ALP1* in activating PcG targets. Gene ontology enrichment analysis suggested that the genes downregulated in *alp1-4* were enriched for a wide range of biological processes particularly those involved in stress response and disease resistance, in contrast to the genes upregulated in *clf-50* which were enriched for ones involved in flower development (S2A and S2B Table). Indeed, when we compared genes downregulated in an *alp1* background with those up-regulated in a *clf* background (in order to identify common targets oppositely regulated) the overlap was small (4 genes, S2C Table) but did include a key PcG target, the floral homeotic gene *APETALA3* (*AP3*). Since the effects of *alp1* mutation are most pronounced in the *clf* mutant background, we also searched for genes that are oppositely regulated by *CLF* and *ALP1* relative to the *clf alp1-4* double mutant background. We identified a small but significant overlap of 12 genes which included the floral homeotic genes *SHATTERPROOF2* (*SHP2*) and *APETALA3* (*AP3*) (S2D Table). Although these results suggested that *ALP1* might play a role in activation of floral homeotic gene expression, we did not observe floral homeotic defects in *alp1* single mutant flowers (Fig 3F). To reveal subtle defects, we removed *ALP1* activity in the weak *leafy-5* (*lfy-5)* mutant background, which has reduced transcriptional activation of floral homeotic genes and is especially sensitive to any mutation that further weakens activation [53]. Indeed, *alp1-4* strongly enhanced *lfy-5* mutations, such that double mutant flowers lacked petals (Fig 3F and 3G); consistent with the enhanced floral phenotype, transcription of *AP3* and *PISTILLATA* (*PI*, like *AP3* is required for petal and stamen specification) was severely reduced in *alp1-4 lfy-5* double mutant inflorescences compared to *lfy-5* single mutants (Fig 3H).

### ALP1 associates with PRC2 *in vivo*

**Fig 4:**
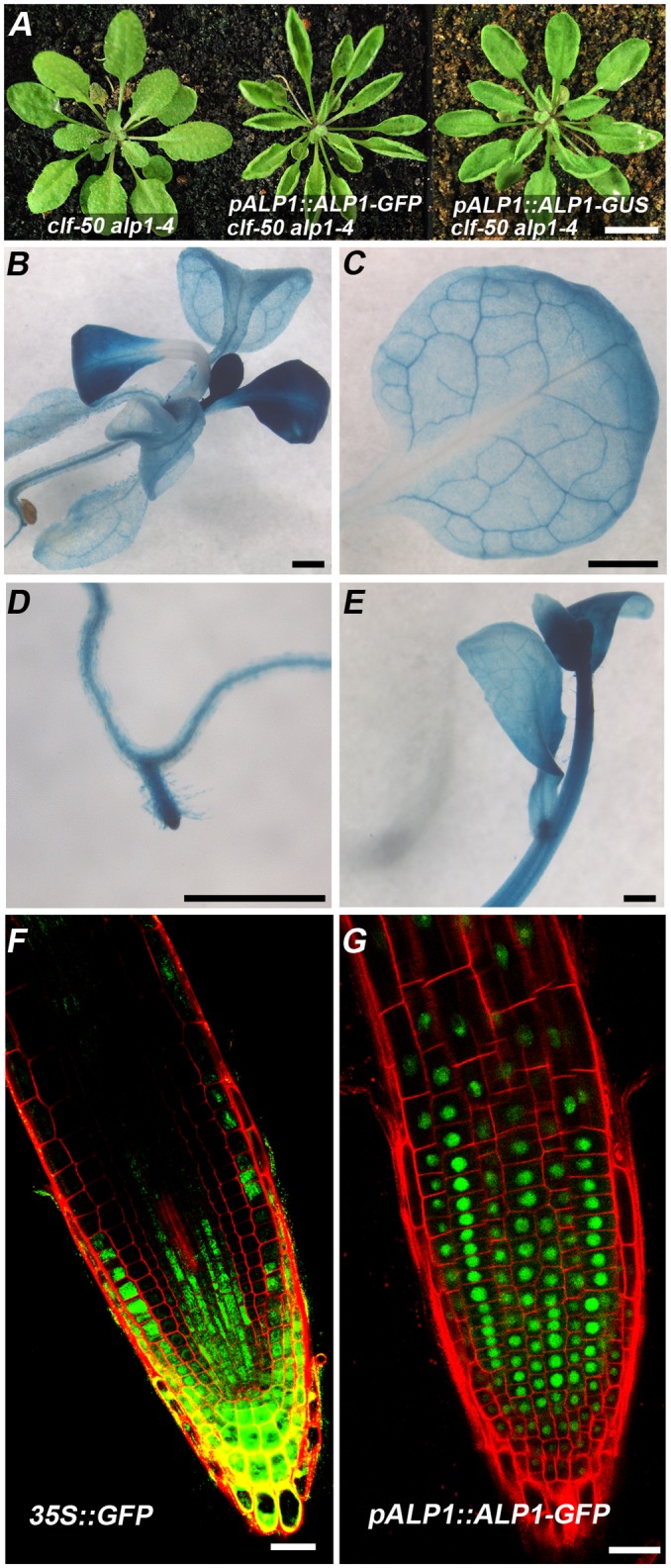
*ALP1* is widely expressed and its protein product is nuclear-localised. (A) Complementation assay in *clf-50 alp1-4* background. The *pALP1*::*ALP1-GFP* transgene fully complements *alp1-4* and restores the clf phenotype, whereas *pALP1*::*ALP1-GUS* gives weaker complementation so that plants retain a partially suppressed clf phenotype. (B-G) Histochemical staining showing *pALP1*::*ALP1-GUS* activity in rosettes (B), leaves (C), roots (D) and inflorescences (E). (F–G) *pALP1*::*ALP1-GFP* is nuclear localised in roots (G), whereas a control *35S*:*GFP* construct shows more diffuse localisation in cytoplasm and nucleus (F). Scale bars are 1cm in A, 1mm in B-E and 20μm in F,G.

RT PCR suggested that *ALP1* was expressed broadly in plants (S1D Fig). To characterise expression further, we made reporters that expressed in-frame fusions of ALP1 with GFP or GUS proteins under control of the native *ALP1* promoter. The GFP fusion construct fully complemented the *alp1-4* mutation whereas the GUS fusion construct only gave a partial complementation (Fig 4A). Since both constructs had the same *ALP1* regulatory sequences, including introns, this suggested that the differences were not in expression but rather in the extent to which the fusions impaired ALP1 protein activity. The *pALP1*::*ALP1-GUS* reporter was expressed broadly in leaves, stems, flowers and roots (Fig 4B–4E), with strongest expression in meristems and young leaves similar to the expression of many other plant chromatin regulators (e.g *CLF* and *SWN*). The ALP1-GFP fusion was nuclear localised in transgenic plants, consistent with ALP1 functioning as a transcriptional regulator (Fig 4F and 4G), and was also widely expressed (S1E Fig). We also made *35S*::*ALP1-GFP* constructs which complemented *alp1-4*, however we did not observe ectopic activation of PcG targets suggesting that ALP1 activity is insufficient to overcome PcG repression. To test whether ALP1 was part of a chromatin-related protein complex, we immunoprecipitated GFP-tagged ALP1 from transgenic plants expressing *pALP1*::*ALP1-GFP* or *p35S*::*ALP1-GFP* constructs and identified co-purifying proteins by mass spectrometry (IP-MS). Strikingly, in both *ALP1-GFP* lines the core PRC2 components SWN, CLF, EMF2, FIE and MSI1 were identified, but not in extracts from *35S*::*GFP* control plants (Table 1). No trxG components were identified in any of the extracts (S3 Table).

**Table 1:** ALP1 co-purifies with Pc-G proteins. The table summarises the results from three independent replicate experiments (IP1-IP2-IP3) and lists the number of uniquely identified peptides from each protein. The total number of peptides identified in each experiment is also shown (all peptides). In IP1 some of the *35S*::*GFP* lysate was lost during filtration, in IP3 there was considerable loss of all samples except *35S*::*GFP-CLF* during the stage tip purification of in gel tryptic digests, hence the lower total number of peptides. The full list of proteins identified is presented as an excel sheet in S3 Table.

Protein	*35S*::*GFP*	*35S*::*GFP-CLF*	*pALP1*::*ALP1-GFP*	*35*::*ALP1-GFP*
**ALP1**	2-0-3	9-13-3	21-24-14	21-25-17
**CLF**	0-0-0	57-70-64	11-15-1	9-14-0
**FIE**	0-0-0	23-27-26	16-21-6	15-17-5
**MSI1**	0-0-0	21-27-25	16-18-5	11-20-5
**EMF2**	0-0-0	27-31-33	21-26-8	16-27-7
**SWN**	0-0-0	0-0-0	27-27-0	18-24-2
**VRN2**	0-0-0	9-12-10	0-0-0	0-0-0
**EMF1**	0-0-0	23-37-17	0-0-0	0-0-0
**LHP1**	0-0-0	9-19-14	0-0-0	0-0-0
**VRN5/VIL1**	0-0-0	20-26-19	0-0-0	0-0-0
**VEL1/VIL2**	0-0-0	27-37-33	0-0-0	0-0-0
**ALL PEPTIDES**	408-1630—818	1623-2714-2045	1663-2210-517	1478-2165-626

We also performed IP-MS on extracts from *35S*::*GFP-CLF* plants (Table 1). Consistent with the presence of CLF in the ALP1-GFP IP, we found ALP1 in the reciprocal GFP-CLF IP, although we also detected a few ALP1 peptides in *35S*::*GFP* controls in two of three replicates. Furthermore, we identified the core PRC2 complex members FIE, MSI1, EMF2, VRN2 and the plant-specific PRC2 accessory components VERNALIZATION5 (VRN5)/VIN3-LIKE1 (VIL1) and VEL1/VIL2 which are thought to boost activity of the HMTase complex [12]. We also found LHP1 which has been variously associated both with PRC1 components and with PRC2 complexes in IP-MS experiments [32,33]. We did not identify SWN, suggesting that PRC2 complexes contain either SWN or CLF as the catalytic component but not both together, consistent with the 1:1 stoichiometry of PRC2 components in structural models [54]. Lastly, we identified EMF1, which has not previously been shown to associate with the PRC2 *in vivo* but has been strongly implicated as a PcG component based on interaction of EMF1 and MSI1 *in vitro* and effects of *emf1* mutation on H3K27me3 levels *in vivo* [28,42]. Strikingly, neither the PRC2 activators VRN5/VIL1 and VEL1/VIL2 nor the alleged PRC1 components LHP1 and EMF1 were present in either the 35S::ALP1-GFP or the pALP1::ALP1-GFP pull-downs suggesting that their presence is mutually exclusive.

**Fig 5:**
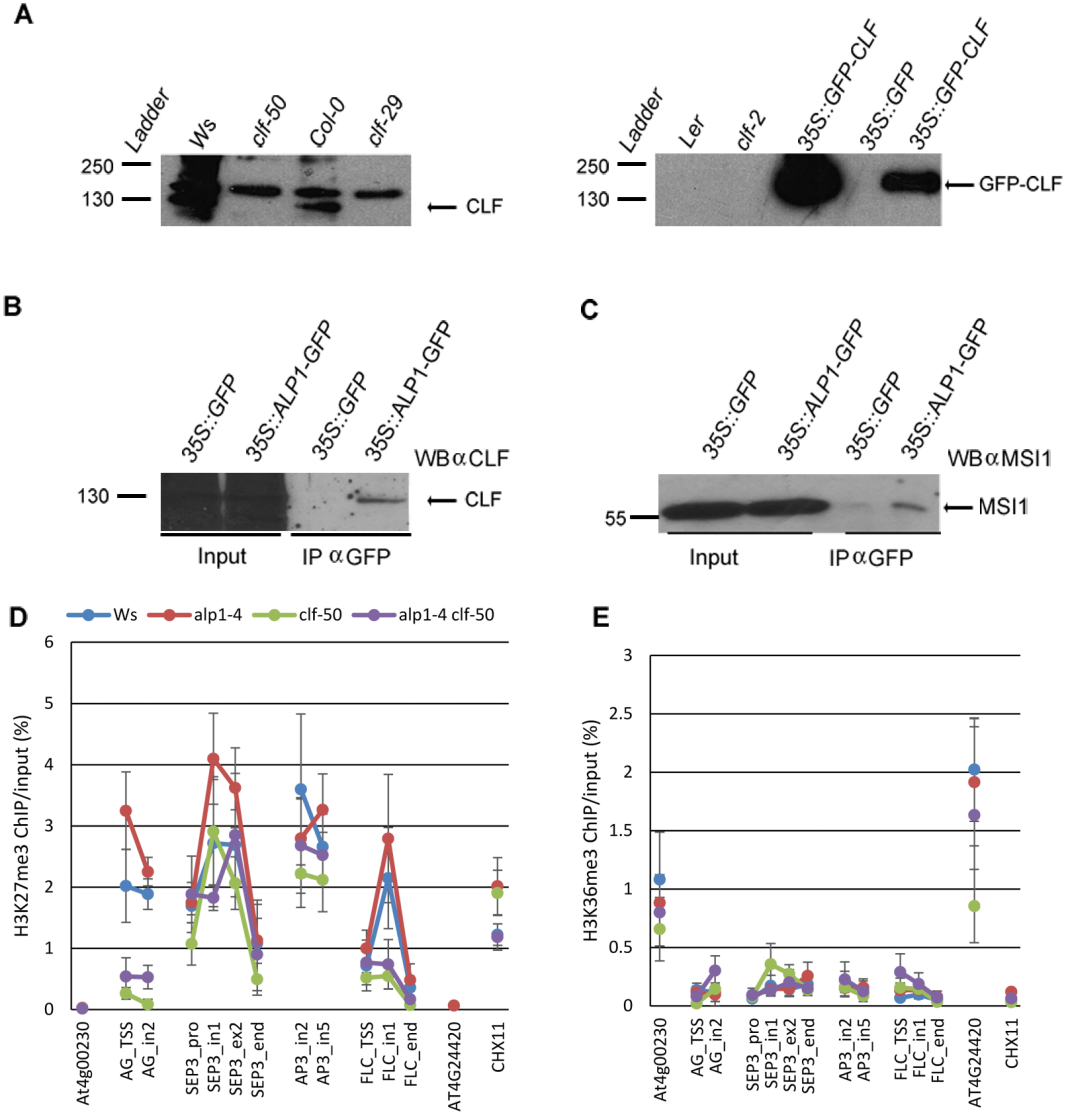
ALP1 interacts with PRC2. (A) Western blot of seedling protein extracts analysed using anti-CLF antibodies. The left and right panels show blots with short (right panel) and longer (left panel) chemiluminescent detection times as the two extracts from *35S*::*GFP-CLF* transgenic plants show much higher expression of GFP-CLF than native CLF. The positions of the size markers in the ladder lane have been marked on the image. Both CLF (≈125kD) and GFP-CLF (≈155 kD) migrate as larger proteins than their predicted sizes (102 and 129 kD, respectively). When the CLF protein was expressed in *E*. *coli* it also migrated larger than predicted, possibly because of the high lysine and arginine content in the N-terminal portion. (B-C) Co-immunoprecipitation experiments in which protein extracts were immunoprecipitated using anti-GFP antibodies, immunoblotted and analysed using anti-CLF (B) or anti-MSI1 (C) antibodies. (D-E) Immunoprecipitation of chromatin prepared from 12-day old Ws, *clf-5*0, *alp1-4* and *clf-50 alp1-4* seedlings using anti-H3K27me3 (D) or anti-H3K36me3 (E) antibodies. Precipitated DNA was quantified using real time PCR and is displayed as percentage of input. PCR fragments were located in promoter (pro), transcriptional start site (TSS), exon (ex), intron (in) and at end of interrogated genes as indicated. Error bars indicate the mean and standard error of three separate experiments, each with three technical replicates. The differences between *alp1* and wild-type or between *alp1 clf* and *clf* were not statistically significant (Tukey multiple comparison of means test) in any of the regions examined.

To verify the association of ALP1 with the PRC2 *in vivo*, we performed co-immunoprecipitation (co-IP) experiments. To make the co-IP assays and IP-MS independent, we immunoprecipitated extracts from *35S*::*ALP1-GFP* plants using different anti-GFP antibodies from those used in IP-MS and analysed the proteins coimmunoprecipitated with ALP1 using Western blotting. To identify CLF we first generated antibodies to an amino-terminal portion of CLF (see S1 File). The antibodies recognised both CLF-GFP and native CLF in western blots of plant protein extracts although they also cross-reacted with other proteins (Fig 5A). Using these antibodies, we confirmed that CLF was co-immunoprecipitated with ALP1-GFP whereas the cross reacting proteins were not (Fig 5B). In addition we verified that MSI1 is co-immunoprecipitated with ALP1 using a well characterised antibody to MSI1 [55] (Fig 5C). Collectively, these results indicate that ALP1 associates with the PRC2 complex *in vivo*. Since *ALP1* is an activator of PcG targets, it presumably antagonises the function of SWN-PRC2 and/or CLF-PRC2. The *clf-50* mutation is a null allele that carries a deletion of the *CLF* locus so that *clf-50* plants have no CLF protein (see Fig 5A) or CLF-PRC2. The most straightforward explanation for the suppression of the clf phenotype by *alp1* mutants is therefore that ALP1 normally inhibits SWN-PRC2 HMTase activity, so that in *clf alp1* mutants SWN-PRC2 inhibition is alleviated allowing it to repress key targets such as *SEP3*. To test this we performed ChIP assays on chromatin at the *SEP3*, *AG*, *AP3* and *FLC* loci (Fig 5D). In *clf-50* H3K27me3 levels were significantly reduced at *AG* and *FLC* intron 1, whereas *SEP3* and *AP3* were less affected. H3K27me3 levels seemed to be increased in *alp1*-*4* compared to Ws and in *alp1*-*4 clf*-*50* compared to *clf*-*50*, but the differences were not statistically significant. Hence, the *alp1* mutation does not alleviate the clf phenotype by restoring H3K27me3 levels. In addition, *alp1* did not affect H3K36me3, consistent with the immunoblot results (Figs 1J and 4E).

## Discussion

Our results offer new perspectives on the organisation of Pc repressing complexes in plants. We confirm that CLF associates with canonical PRC2 members *in vivo*, but surprisingly also with several other PcG members including EMF1, hitherto thought to be in the PRC1 complex. Most strikingly we find that an ancient domesticated tranposase is a component of PRC2 complexes and antagonises their function in gene repression. This is the first example of a domesticated transposase becoming part of the core epigenetic machinery of the host and raises the question of whether the association evolved to benefit transposons or the host.

### CLF associates with both PRC2 and PRC1 components

Previous IP-MS experiments using tagged versions of the core PRC2 components EMF2 or MSI1, or the accessory component VRN5/VIL1 identified PRC2 complexes containing SWN but not CLF as the catalytic unit [12,33]. Using tagged CLF we identified EMF2 and VRN2 [Su(z)12 homologues], MSI1 (Nurf55 homologue), and FIE (Esc homologue), confirming that CLF occurs in both VRN2-PRC2 and EMF2-PRC2 complexes *in vivo*. The discrepancy between these results from reciprocal pull down experiments might be explained if SWN is more abundant or more stable than CLF *in vivo*, so that the bulk of EMF2/MSI1 containing complexes have SWN rather than CLF. In addition, we found VRN5/VIL1 and VEL1/VIL2, two related PHD domain proteins that have also been shown to associate with the core PRC2 in pull downs of MSI1 or of VIL1 itself [12,33]. This suggests that VIL1 and/or VIL2 are components of most CLF-PRC2 complexes. No mutant phenotype has been reported for *vil2* mutants, whereas *vil1* mutants have an impaired vernalization response broadly similar to that of *vrn2* mutants indicating that *VIL1* is needed for full activity of VRN2-PRC2 [56,57]. In the absence of vernalization, *vil1* mutants have a very weak phenotype relative to *clf* mutants, but it is possible that more severe defects are masked by redundancy between *VIL1* and *VIL2* [56]. Thus VIL1 and VIL2 are likely to be required for the full activity of the CLF-PRC2.

Additional to the core and accessory PRC2 components, CLF also pulled down two proteins—LHP1 and EMF1—generally thought to be in plant PRC1-like complexes [25]. LHP1 and EMF1 are functional equivalents of Drosophila Pc and Psc, and have been found to interact with each other as well as with the plant homologues of the other Drosphila core PRC1 components, namely the AtBMI1 and AtRING1 proteins [29,31,42]. The fact that CLF pulls down the core PRC2 together with EMF1 and LHP1 does not prove that all are in the same complex, as similar results would be obtained if there are distinct CLF-PRC2 and CLF/EMF1/LHP1 complexes. However, there is additional evidence to support EMF1 and LHP1 associating with the other PRC2 components. Notably, IP-MS experiments using MSI1 identified LHP1 (but only when cross-linked protein extracts were used) and LHP1 was found to co-immunoprecipitate both with MSI1 and also with EMF2 [33]. Furthermore, both EMF1 and LHP1 directly interact with MSI1 in *in vitro* pull down assays [28,33]. One possibility is that in plants a PRC1-like complex (AtRING1/AtBMI1/LHP1/EMF1) interacts with a CLF-PRC2 complex via its MSI1 component. This would be consistent with recent proteomic studies using cross-linked extracts, which suggest that in *Drosophila* the PRC1 and PRC2 complexes can interact via a common bridging component, Sex Combs on Midleg (Scm) [58]. Alternatively, EMF1 and LHP1 may participate in distinct complexes, namely a PRC1-like complex (AtRING1/AtBMI1/EMF1/LHP1) with a role in histone ubiquitination (via AtRING1/AtBMI1) and in a PRC2/EMF1/LHP1 complex with a role in histone methylation and transcriptional silencing (via the EMF1 component). The latter scenario is more consistent with the fact that no AtBMI1 or AtRING1 proteins were found in the CLF IP-MS and also with genetic data suggesting that *AtRING1* and *AtBMI1* genes regulate only a subset of PcG targets in *Arabidopsis*. Further biochemical purification of plant PcG complexes, together with *in vitro* reconstitution experiments should help distinguish between these alternatives.

### ALP1 associates with the core PRC2 but not PRC1

Using two different transgenic lines (expressing ALP1-GFP from the native or the *35S* promoter) and three independent experiments (effectively six replicates) we unequivocally identify the core PRC2 components FIE, MSI1, EMF2, SWN and CLF as ALP1 partners *in vivo*. PRC2 complexes contain a single catalytic unit, here either SWN or CLF but never both proteins, as can be seen from the fact that SWN was not found in the CLF IP-MS. Since ALP1 IP-MS retrieves SWN and CLF, ALP1 interacts both with SWN-PRC2 and CLF-PRC2 complexes *in vivo*. We identified EMF2 but not VRN2, which may indicate a preference for EMF2 over VRN2 containing complexes however genetic data suggests EMF2-PRC2 is more abundant in the absence of vernalization treatment. Notably, we did not identify any peptides from VIL1, VIL2, EMF1 or LHP1 in any of these experiments. Given that all of these were identified with high confidence in all three IP-MS experiments using CLF, we conclude that ALP1 associates with a subset of CLF and SWN-PRC2 complexes that lack VIL1/VIL2/EMF1/LHP1.

### *ALP1* antagonises the PcG

Genetically *ALP1* has all the hallmarks of a trxG gene. Firstly, *alp1* mutants suppresses the phenotype of several PcG mutants. Transcriptional profiling showed that this is because when PcG activity is impaired, ALP1 activity is needed to activate the bulk of the target genes that are normally de-repressed. Secondly, even when PcG are fully active, *ALP1* has a role in overcoming PcG repression at some PcG targets. This is revealed by subtle defects in the transcriptional activation of the floral homeotic genes *AP3* and *PI* in *alp1* mutants, but also in that a significantly higher proportion of the genes downregulated in the *alp1* background are PcG targets than in the genome as a whole. Thirdly, *alp1* mutants enhance the phenotype of several trxG mutants including *ult1*, *ult2* and *efs*. Interpretation of these synergistic interactions is complicated as there may be substantial genetic redundancy (for example, EFS is not the only *Arabidopsis* H3K36 HMTase), but the simplest explanation is that *ALP1* acts in parallel to *ULT1/2* and *EFS* in opposing PcG repression.

The finding that a protein inhibiting PcG silencing is actually a component of CLF and SWN-PRC2 complexes is counter-intuitive. One possibility is that the ALP1 containing PRC2 complexes constitute a small specific fraction of the total PRC2 and occur at situations where PcG repression is being downregulated or over-turned. This is supported by the finding that whereas CLF and SWN are readily detected in ALP1 IP-MS, in the reciprocal experiment involving CLF IP-MS, ALP1 is not greatly enriched over background—in other words, most or all ALP1 occurs in PRC2 complexes, whereas a much smaller fraction of CLF-PRC2 contain ALP1. A comparable example of an inhibitor interacting with PRC2 was recently described, in which the tumor suppressor BRCA1 interacts with PRC2 in mouse embryonic stem cells and inhibits PRC2 binding to genes involved in cell differentiation, promoting their expression [59].

Under our growth conditions, *alp1* single mutants did not show major developmental phenotypes, and did not affect the expression of most of the genes mis-regulated in *clf* mutants. Thus *ALP1* regulates a small subset of PcG targets under laboratory conditions. However, it was notable that the genes that were downregulated in *alp1* were enriched for functions in disease resistance and stress response. An intriguing possibility is that *ALP1* may be required to overcome PcG silencing of genes involved in stress or disease, and therefore *alp1* mutants may show more severe mutant phenotypes under other growth conditions closer to natural environments.

### How does ALP1 inhibit PcG action?

**Fig 6:**
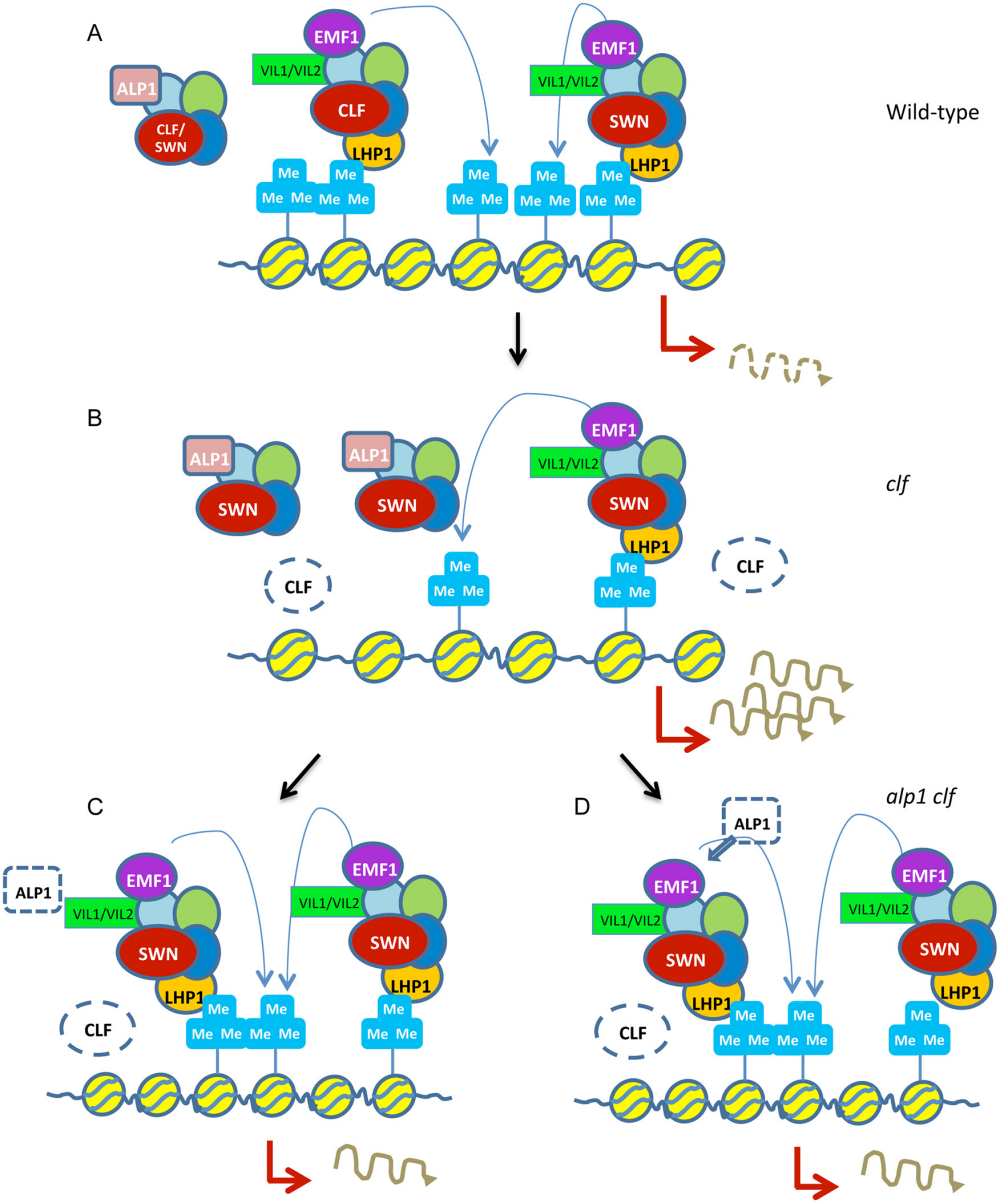
Alternative models for how ALP1 antagonises PcG function. (A) In wild type, both SWN-PRC2 and CLF-PRC2 are present and PcG targets are heavily H3K27me3 decorated. Most PRC2 complexes are fully active (blue arrow) but a fraction associate with ALP1 and are inhibited. Transcription is inhibited through the action of EMF1, which associates with the PRC2. (B) In *clf* mutants, there is a reduced amount of total PRC2 as only SWN-PRC2 is present, leading to reduced H3K27me3 at some targets and transcriptional derepression. A larger fraction of total PRC2 associates with ALP1 and is inhibited. (C) If ALP1 inhibits the H3K27me3 HMTase activity of SWN-PRC2, for example by impairing its interaction with VIL1 and VIL2, then in *alp1 clf* double mutants H3K27me3 levels or spread increase at targets, partially restoring transcriptional silencing. (D) If ALP1 inhibits the interaction of EMF1 with SWN-PRC2, in *alp1 clf* double mutants more SWN-PRC2 is associated with EMF1, leading to increased transcriptional silencing without an increase in H3K27me3.

It is notable that *alp1* mutations can only suppress relatively weak PcG mutants (*lhp1* and *clf*) in which PRC2 activity is impaired but not abolished. *clf swn* mutants, which lack all sporophytic PRC2 activity, were not rescued by *alp1*, implying that PRC2 activity is needed for rescue. The simplest explanation for the suppression of *clf* and *lhp1* by ALP1 is that ALP1 inhibits the HMTase activity of the CLF-PRC2 and SWN-PRC2. Indeed, by blocking the association with accessory components such as VIL1 and VIL2, ALP1 is likely to reduce HMTase activity. In *clf* mutants H3K27me3 levels are reduced at some targets, but if the HMTase activity of SWN-PRC2 (or in *lhp1* mutant backgrounds, both CLF-PRC2 and SWN-PRC2) is upregulated when *ALP1* activity is withdrawn normal H3K27me3 levels and silencing might be restored (Fig 6A–6C). Additionally, if ALP1 possesses DNA binding activity it may inhibit silencing indirectly by luring the PRC2 away from PcG targets to other sites in the genome. Against these scenarios, our H3K27me3 ChIP experiments did not support an increase in H3K27me3 at PcG targets in *alp1* mutants. Given that *alp1* mutants give a weak rescue of PcG mutant phenotypes, and that subtle effects on H3K27me3 levels may only be visible in dividing cells rather than whole seedlings [e.g. see 33] we can’t exclude that *ALP1* inhibits PRC2 HMTase activity and it will be important to test the effects of ALP1 on PRC2 catalytic activity *in vitro*. An alternative possibility is that ALP1 acts by inhibiting a function of the PRC2 independent of its H3K27me3 HMTase activity, for example a direct role in silencing transcription. Notably, we found that CLF-PRC2 but not ALP1-PRC2 associates with EMF1, a protein playing a similar role to the *Drosophila* PcG protein Psc in inhibiting chromatin remodeling [27] and transcription *in vitro*. If ALP1 competes with EMF1 for CLF- and SWN-PRC2, then removing ALP1 activity might restore silencing by increasing EMF1 occupancy at *CLF* targets (Fig 6D). Indeed, *alp1* did not rescue *emf1-1* mutants, suggesting that EMF1 activity is important for ALP1 to rescue PcG.

### Why is a domesticated transposase a PRC2 component?

Although there are numerous examples of genes which have arisen by domestication of transposases [60], to our knowledge this is the first case where a domesticated transposase has become an inhibitory component of the host core epigenetic machinery. Autonomous *PIF/Harbinger* transposons are known to mobilise a group of small non-autonomous transposable elements (specifically the *Tourist* class of Miniature Inverted repeat Transposable Elements [MITEs]) that have proliferated massively within plant genomes—for example, there are around 90,000 MITEs in the rice genome, comprising the bulk of the transposon content [50]. A further characteristic feature of MITEs is that they have a strong preference to insert into single copy, euchromatic regions of the genome [46,50]. Plant hosts typically inactivate transposons by siRNA mediated DNA methylation, which silences expression of their transposase [for review see 61]. In several cases, transposons have been shown to encode proteins that inhibit the host machinery mediating their methylation [62,63], rather as plant viruses encode anti-silencing proteins that interfere with the siRNA machinery. Although PcG silencing is typically thought of in terms of developmental target genes, it also serves a backup function in silencing transposons when DNA methylation is compromised. Thus, in *met1* mutants where CG DNA methylation is severely reduced, there is a massive relocation of H3K27me3 onto transposons [64]. Similarly, in endosperm tissue where DNA methylation levels are generally low, transposons are frequently H3K27me3 methylated and this contributes to their transcriptional silencing [65]. Furthermore, studies using the unicellular green alga *Chlamydomonas rheinhardtii* suggest that the ancestral role of PRC2 in may have been in silencing transposons and other repetitive elements [66]. One possibility therefore is that the association of a transposase with the PRC2 originally evolved as a way for *PIF/Harbinger* transposons to evade host surveillance and promote their own proliferation. This would be particularly effective if *PIF/Harbinger* transposons have also evolved means to inhibit host RNA-directed DNA methylation systems. An association with PcG would also benefit the tranposons by targetting them to euchromatic gene rich regions of the genome, where it may be difficult for the host to permanently silence the transpsoson due to effects on expression of neighbouring genes.

Alternatively, the association of a domesticated transposase with the PRC2 has arisen because it benefits the host. Given that *ALP1* is an ancient gene in land plants, it is highly unlikely that it would have been conserved if it functioned solely to promote *PIF/Harbinger* transposon proliferation. This would require *ALP1* to be part of an active transposon, able to proliferate faster than its hosts could eliminate it, whereas *ALP1* is 1–2 copy and immobile. In many cases where transposases have been domesticated, the DNA binding property of the transposon has been conserved, rather than the endonuclease activity [60]. However, in *PIF/Harbinger* this activity is encoded by a second gene which encodes a Myb class DNA binding protein that is necessary for transposition and has been shown to bind DNA sequences at the tranposon ends and to interact with the nuclease protein to form a functional transposase [67,68]. ALP1 is unlikely to retain nuclease activity, as studies expressing the rice *PIF/Harbinger* class transposon *PING* in a heterologous system have demonstrated that mutating just one of the three residues in the DDE triad drastically reduces its ability to catalyse transposition [69]. However, it is possible that it retains the ability to interact with a Myb class DNA binding protein and this is useful for targetting PcG to its targets. This would be comparable to vertebrates, where the nuclease of *Harbinger* has been domesticated to produce the *Harbi1* gene and the Myb gene to produce the *Naif1*. Although the biological function of these genes is unknown, the HARBI1 and NAIF1 proteins are able to interact [67]. A role for Myb proteins in PcG recruitment to targets in plants has also been demonstrated [70]. A role for ALP1 in recruitment does not however explain why it antagonises PcG silencing. A notable feature of transposons is that they are often activated during stress—for example, in *Arabidopsis* several retrotransposons are activated by heat shock treatments[71,72], and in blood orange varieties, anthocyanin production is stimulated by a cold-inducible retrotransposon inserted upstream of the *RUBY* gene [73]. The inhibitory interaction of ALP1 with the PcG might have arisen as a way for the plant to promote the activation of PcG target genes involved in stress response. This would be consistent with the fact that ALP1 targets are enriched for genes involved in biotic and abiotic stress response. By disabling the nuclease activity of the transposase, the plant host may also have limited the side effect of promoting transposon proliferation. It will be interesting in future to test the role of *ALP1* in transposon mobilisation and stress response, and also to see whether the vertebrate *HARBI1* and/or *NAIF* domesticates have any role in epigenetic control by PcG or DNA methylation.

## Supporting Information

S1 File Supplemental Experimental Procedures. (DOCX)

S1 Fig *ALP1* gene structure and expression. (A) *ALP1* gene structure showing the position of the lesions in four independent alleles. Exons are shown as boxes, introns as lines, T-DNA insertion as a triangle. The *alp1-3* allele (CSHL ET1398) harbours a modified *Ds* transposon insertion. Molecular analysis of *alp1-4* revealed that the T-DNA insert is complex, containing at least two T-DNA copies in inverted orientation and a 62 bp deletion of *ALP1* sequences flanking the insert; however there were no major rearrangements of the *ALP1* locus. (B) *alp1-3* partially suppresses the null *clf-2* mutation, particularly in short days (SD). Plants were 26 days old (LD) or 51 days old (SD). Scale bar 1cm. (C) Double mutant between *alp1-1* and *at3g55350* (Salk_122829). Plants grown in long days. There was no obvious difference in the rosette, floral phenotype, or flowering time. Plants shown are siblings in progeny of an *alp1-1* individual heterozygous for the T DNA insertion allele at *at3g55350*. (D) RT PCR analysis of *ALP1* expression in different tissues. YL, young leaves; AL, adult leaves; B, flower buds; F,flowers; S, seedlings; R, roots. *EiF4A* is a reference gene used to normalise cDNA amount used in each experiment. (E) Western blot analysis of the presence of ALP1-GFP in tissues. Total crude proteins wereextracted from a variety of tissues including roots (R), inflorescence stems (S), 2-week-old seedlings (2w), rosette leaves (Ro), cauline leaves (C), flower bud and inflorescence (F) and siliques (S) of transgenic *pALP1*::*ALP1-GFP alp1-4* plants, and then analysed by Western blotting using a mouse monoclonal antibody against GFP. Protein extracts from Ws and *pLHP1*::*LHP1-GFP* (+) were also included as negative and positive control, respectively.
(TIF)

S2 Fig Double mutants of *alp1-4* and *mea-emb173* or *atx1-1*. (A) Seed from *mea-emb173/+* plants (left panel) and *mea-emb173/+ alp1-4* plants (right panel). Both plants segregate lighter coloured plump, seed and darker coloured collapsed seed due to the zygotic lethality of maternally inherited *mea-emb173*. Scale bar 1mm. (B) Double mutants of *alp1-4 atx1-1*, long day grown plants. The *alp1-4 atx1-1* double mutant does not enhance the mild atx1 phenotype. Scale bar 1 cm. (C) Floral phenotypes. Flowers of the double mutants were similar to those of the *atx1-1* single mutant with no obvious enhancement. Scale bar 500 μm.
(TIF)

S3 Fig Genetic interactions between *ALP1*, *ULT1* and *EFS*. (A) The flowers and siliques of *alp1-3* and *ult1-1* mutants. The silique of *alp1-3 ult1-1* was composed of four carpels, while in *ult1-1*, it was usually three. The *ult1-1* and *alp1-3 ult1-1* flowers typically had extra petals relative to wild-type. Scale bars, upper panel, 1 mm; lower panel, 0.5 mm. (B) Statistical analysis of floral organ numbers in *alp1-3* and *ult1-1* mutants. The floral organs of the initial 10 flowers on primary inflorescence stems were counted and the average numbers of each floral organ are shown with 1 standard error of the mean as error bars. Data were collected from 11–19 individual plants. The stars mark the data that are significantly different from data of wild-type plants in one way ANOVA tests (p<0.001). Note that there was also a significant difference between *alp1-3 ult1-1* and *ult1-1* (p<0.001). (C) Double mutants between *alp1-1* and *efs* (Salk_026442, also known as sdg8-2) in uniform Col-0 background. The double mutants were much smaller and more dwarved than the single mutants.
(TIF)

S4 Fig The *alp1-3* mutation enhances the *ult2-2* floral phenotype. (A) The flower of *alp1-3* and *ult2-2* mutants. In *ult2-2* and *alp1-3*, the numbers of floral organs are normal, whereas the double mutant *alp1-3 ult2-2* displayed extra petals. Photographs were taken under the same scale. (B) Statistical analysis of floral organ numbers in *alp1-3* and *ult2-2* mutants. The floral organs of initial 10 flowers on primary inflorescence stems were counted and the average numbers of each floral organ are shown with 1 standard error of the mean as error bars. Data were collected from 11–19 individual plants. The stars mark the data that are significantly different compared with data of wild type plants (p<0.001, ANOVA test).
(TIF)

S5 Fig Alignment of land plant ALP1 homologues and transposases. Alignment between selected land plant ALP1 proteins, rice Pong transposase and mouse Harbi1 nuclease made using MUSCLE. Amino acids are shaded according to the RasMol colour scheme based on their properties. Black lines underneath the alignment indicate six regions previously found to be conserved between PIF/Harbinger nucleases [1], the red line a large region of conservation between plant PONG transposases [2]. The black boxes indicate the position of the DDE catalytic triad that is conserved amongst tranposases. Analysis of the Arabidopsis ALP1 protein sequence using the structural prediction program PHYRE [3] identified a potential helix turn helix turn helix motif with low similarity to the DNA binding domain of homeodomain class proteins. The position of the helices is indicated in green above the alignment. The sequence identities are as described in the legend to Fig 2, mouse Harbi1 is Genbank GI:154759331.
(TIF)

S6 Fig *ALP1* is in a syntenic region in *Arabidopsis* and several other eudicot species. (A) Comparison of the genomic region around *ALP1* in *Arabidopsis thaliana* with corresponding regions in *Arabidopsis lyrata* and *Populus trichocarpa*. The genes neighbouring *ALP1*, their orientation and relative order are conserved between the three species, indicating that *ALP1* has not transposed at least in the time since these species diverged from their common ancestor. Futher manual inspection confirmed that the genes neighbouring *Populus trichocarpa ALP1* on LGII retrieve the genes neighbouring *ALP1* in *Arabidopsis* as best hits in reciprocal TBLASTN searches. (B) Intron position is conserved between *ALP1* and *At3g55350* genes in various angiosperm species. The red arrow indicates the position at which the intron interrupts the predicted protein sequences of the different genes. The alignment of a portion of the protein sequences indicates that the intron is at the same position in all genes, strongly suggesting a common evolutionary origin for *ALP1* and *At3g55350*. With the exception of *Arabidopsis ALP1* which contains two introns, all the other genes contain a single intron.
(TIF)

S1 Table RNA seq data. Excel file with multiple sheets. Sheet one is the Raw read data for RNA seq comparisons of Ws, *clf-50*, *alp1-4* and *clf-50 alp1-4* 10 day old seedlings grown in long days in tissue culture plates. Sheets two to six show genes significantly mis-regulated in the various pairwise comparisons.
(XLSX)

S2 Table Common targets of ALP1 and CLF. (A) Comparison of genes upregulated in *clf-50* vs Ws (331 genes) and down-regulated in comparision of *alp1-4* with Ws. (126 genes). Only four genes were common to both sets, an overlap which is not statistically significant at 5% level (p = 0.0697471, hypergeometric test). (B) Comparison of genes upregulated in comparison of *alp1-4 clf-50* vs *alp1-4* (210 genes) with genes down-regulated in comparison of *alp1-4 clf-50* with *clf-50* (223 genes). Twelve genes were common to both sets, a highly significant overlap (p = 1.989284e-07 hypergeometric test)
(XLS)

S3 Table Full data set for IP-MS proteomic experiments. Excel sheet summarising the IP-MS data. Three separate sheets representing the three biological replicates. The numbers refer to uniquely identified peptides.
(XLSX)

S4 Table Oligonucleotide primers. Excel sheet with list of oligonucleotide primers used.
(XLSX)

S5 Table Genetic materials used. Origin of the different genetic materials used including nature of the mutation and the genetic background in which the mutant was isolated.
(DOCX)
