# Correction: Kicking against the PRCs - A Domesticated Transposase Antagonises Silencing Mediated by Polycomb Group Proteins and Is an Accessory Component of Polycomb Repressive Complex 2

**DOI:** 10.1371/journal.pgen.1005812

**Published:** 2016-02-19

**Authors:** Shih Chieh Liang, Ben Hartwig, Pumi Perera, Santiago Mora-García, Erica de Leau, Harry Thornton, Flavia de Lima Alves, Juri Rappsilber, Suxin Yang, Geo Velikkakam James, Korbinian Schneeberger, E. Jean Finnegan, Franziska Turck, Justin Goodrich

The seventh and eighth authors’ names are spelled incorrectly. The correct names are: Flavia de Lima Alves and Juri Rappsilber.

The authors contributions with the corrected initials are provide here:

Conceived and designed the experiments: JG FT SCL BH JR. Performed the experiments: SCL BH PP FdLA SMG JG SY HT EdL. Analyzed the data: FdLA KS GVJ JG FT. Wrote the paper: JG FT EJF BH SCL

The 7th author’s current address is missing. Flavia de Lima Alves’ current address is: Centre for Microbial Chemical Biology, Department of Biochemistry, McMaster University, Ontario, Canada

The affiliation for the 8th author is incomplete. Juri Rappsilber is affiliated with: Department of Bioanalytics, Institute of Biotechnology, Technische Universität Berlin, Berlin, Germany in addition to the listed affiliation: 3. Wellcome Trust Centre for Cell Biology, University of Edinburgh, Edinburgh, United Kingdom

The following information is missing from the Funding section: The Wellcome Trust generously funded this work through a Senior Research Fellowship to JR (103139), a Centre core grant (092076) and an instrument grant (108504).
