# Supplementary material for: Kicking against the PRCs – A Domesticated Transposase Antagonises Silencing Mediated by Polycomb Group Proteins and Is an Accessory Component of Polycomb Repressive Complex 2
Source: PLoS Genet. 2015 Dec 7;11(12):e1005660. doi: 10.1371/journal.pgen.1005660 (PMC4671723; doi:10.1371/journal.pgen.1005660)
Supplement: S1 File — (DOCX) [file pgen.1005660.s001.docx]

**Supplemental Experimental Procedures**

*Growth conditions and plant materials*

Seeds were sown on soil directly or in tissue culture on Murashige-Skoog (MS) agar (0.64 g MS salts [Duchefa] in 1 L of water containing 0.9% w/v agar, 0.3% sucrose, pH 5.7) plates after sterilization and then cultured in growth chambers under fluorescent light. The day/night temperature was 22^o^C/20^o^C. Long day conditions (LD) were 16 hours light and 8 hours dark cycles, whereas short day (SD) conditions were 8 hours of light and 16 hours of dark.

The T-DNA mutagenesis screening and the method for recovering the T-DNA insertion site were described previously [4]. The origin and genetic backgrounds of the mutant lines used are summarised in S5 Table.

Total RNA extraction and gene expression analysis

Total RNA was extracted from 0.05 - 0.1 g of plant material. Tissue was ground in liquid nitrogen and 1 ml Trizol (Invitrogen) was added to the powder and mixed by vortexing. The mixture was centrifuged for 10 minutes (min) at 16,000 g at 4^o^C. The supernatant was transferred to a new tube and 200 µl of chloroform was added. After mixing by vortexing, the milky solution was centrifuged for 10 min at 16,000 g and the clear upper liquid was transferred into a new tube. 0.25ml of 1.2M sodium chloride 0.8M sodium citrate (pH 5.5), and 0.25 ml isopropanol were added and mixed well. Then the solution was stored at -20^o^C for 20 min to help RNA precipitation. A white pellet of RNA was formed after centrifugation for 10 min at 16,000 g at 4^o^C. The pellet was washed with 1 ml of 70% ethanol in DEPC treated H_2_O, air dried then dissolved in 50 µl DEPC treated H_2_O and stored at -80^o^C. First strand cDNA was synthesised using 1 µg of total RNA primed with 1 µl of 100 µM anchored oligo dT primer (5’-TTTTTTTTTTTTTTTTTTTTV-3’) and DEPC-treated H_2_O in a final volume of 10 µl. The mixture was heated to 65^o^C for 10 min and immediately cooled on ice. The reverse transcription reaction was performed in a 20 μl volume containing 10 U RNase inhibitor (Promega), 100 µM DTT, 10 mM dNTP, 1× MMLV buffer (Promega) and 100 U of MMLV Reverse Transcriptase (Promega). The reaction was incubated at 25^o^C for 30 min, followed by 42^o^C for 90 min, and finally at 70^o^C for 10 min. The final cDNA product was diluted to 200 μl in DEPC-treated H_2_O and stored at -20^o^C. Quantification of gene expression by real time PCR was performed using a LightCycler480 (Roche). PCR reactions contained 1X SYBR master premix (Roche), 5 μl cDNA and 200 μM primers in a 15 μl total volume. The amplification program comprised an initial 3 minutes at 95°C followed by 50 cycles of denaturation at 95°C for 15 seconds, annealing at 55°C for 15 sec, and finally extension/detection at 72°C for 15 sec. Three biological replicates were made for each sample. Data analysis was performed using the 2nd derivative max method in the LightCycler480 (Roche) software. For each primer set, the amplification efficiency was calculated by analysis of a calibration curve made using a 1 – 10^-3^ dilution series of cDNA again using the supplied software. The crossing point (Cp) values were converted to relative expression or enrichment levels manually in Excel. Gene expression was normalised relative to the *EiF4A* reference gene.

RNA-seq analysis

Total RNA was extracted (as above) from 10 day old seedlings grown on tissue culture plates in LD; three biological replicates were made for each sample. The RNA samples were processed by the Max Planck Genome Center Cologne for Illumina True-seq sequencing. All individual samples were barcoded and sequenced to a total of 63 million reads. We used SHORE V0.6 mRNA application, to count the raw number of reads per transcript [21]. A maximum of one mismatch in barcode was allowed to assign reads to respective samples. Reads from each samples were separately aligned back to Arabidopsis cDNA (TAIR 10, http://www.arabidopsis.org/) using Genomemapper allowing a maximum of two mismatches [22]. The aligned reads were converted to read counts per transcript using SHORE count function. For the expression analysis, the edgeR package was used [23]. We calculated the normalization factors using the function calcNormFactors, which correct for the different compositions of the samples. The read count per gene in samples was adjusted using normalization factor and common dispersion was calculated using function estimateCommonDisp. Pairwise tests were performed between each group using the function exactTest and differentially expressed genes were extracted using toptag function.

Generation of transgenic lines

The *ALP1* genomic and cDNA sequences were amplified by PCR using a Phusion proofreading polymerase (NEB) according to the manufacturer’s instructions and the resulting products were cloned using pENTR/D-TOPO plasmid (Invitrogen). The resulting clones were sequenced to confirm that no mutations were introduced during amplification. The sequences of primers used are given in Supplementary Table 4. The cloned *ALP1* fragments were transferred into destination binary vectors pGWB1, pGWB3, pGWB4 or pGWB5 [24] by Gateway recombination using LR clonase (Invitrogen) following manufacturer’s instructions.

β-glucuronidase (GUS) staining

Plant material was harvested and incubated for 30 min in 90% v/v acetone on ice in order to permeabilise tissue. Before the tissue was stained for activity of the *GUS* reporter gene, acetone was washed out by rinsing in distilled water (dH_2_O). Then tissue was immersed in GUS staining solution (0.05 mM NaHPO_4_, pH 7.2, 0.5 mM K_3_Fe(CN)_6_, 0.5 mM K_4_Fe(CN)_6_, 1% Triton X-100, 2 mM 5-Bromo-4-chloro-3-indolyl β-D-glucuronide [X-GlucA]) while a vacuum was applied for 10 min twice to facilitate incorporation of the substrate for the GUS gene product. The tissue was subsequently incubated at 37°C overnight. After the staining solution was removed, the stained tissue was washed with 70% ethanol (EtOH) for 1 hr to remove chlorophyll, followed by 85% EtOH for 1 hr and 95% EtOH for 1 hr. The stained tissue was stored in 95% EtOH at room temperature.

Confocal microscopy

To analyse GFP fluorescence in transgenic plants carrying GFP reporter gene constructs, freshly harvested roots of 10-day-old seedlings from MS plates were counterstained with 5 μg/ml propidium iodide (PI) by immersion in the solution for 5 minutes. The roots were detached from hypocotyls, placed on microscope slides, covered with cover slides, and observed on an Olympus Fluoview confocal microscope, using filter sets for texas red and GFP fluorescence.

Western blot analysis

Proteins were extracted by boiling 0.1 g of plant material in 200 μl 2X Laemmli buffer (0.125 mM Tris, pH 6.8, 4% w/v SDS, 18% glycerol, 0.024% w/v bromphenol-blue, 1.43 M β–mercaptoethanol, 0.2% protease inhibitor cocktail [Sigma]). After boiling for 10 min, the insoluble part was removed by centrifugation, and the supernatant was loaded on to polyacrylamide gels to be separated by sodium dodecyl sulfate polyacrylamide gel electrophoresis (SDS-PAGE). Separated proteins were then transferred to a nitrocellulose membrane (0.22 μm pore, Whatman). Before incubating the membrane with the antibody solution, the membrane was blocked with 10% w/v skimmed milk in 1X phosphate buffered saline [PBS, 137 mM NaCl, 2.7 mM KCl, 10 mM Na_2_HPO_4_ • 2 H_2_O, 2 mM KH_2_PO_4_, pH=7.4], containing 0.1% Tween-20 (PBST) for 1 hr at room temperature. The primary antibody was diluted in blocking buffer (1% skimmed milk in 0.1% PBST) roughly 1:1000~1:5000 depending on the antibody used. For anti-GFP (Roche 11814460001), 1:1000 was used. The membrane was incubated in the primary antibody solution at 4°C overnight with gentle agitation (20 rpm). Then the membrane was incubated with the secondary antibody (typically a mouse monoclonal antibody against rabbit IgG, conjugated to horseradish peroxidase, HRP) diluted (usually 1:5000) in blocking buffer. The bound antibodies were detected by chemiluminescence using a kit (Pierce® Western Blotting substrate, #32109). Once the ECL solutions were applied, the signal was exposed to X-ray films. The films were developed using an automatic developing machine (Konica SR101A).

Chromatin immunoprecipitation (ChIP)

ChIP was performed as previously described [25] using α-H3K27me3 [Millipore 07-449], and α-H3K36me3 (Diagenode pAb-058-050) antibodies.

Immunoprecipitation (IP) for mass spectrometry and co-IP

For the IP-MS we followed the procedure described in [26] with minor modifications. Protein extracts were made from 2-5g inflorescence tissue harvested from plants grown at 16^0^C (to increase the size of inflorescences). The entire experiment was replicated on three separate occasions. Tissue was frozen in liquid nitrogen, finely ground using pestle and mortar, and resuspended in 15ml IP buffer (10mM Tris pH 7.5, 150 mM NaCL, 0.5% Igepal, 1% Triton) containing 1X protease inhibitor cocktail (Roche) and 0.1mM PMSF or 1mM Pefabloc (Roche). Proteins were extracted by incubation with gentle rotation at 4^o^C for 30 minutes and the extracts were cleared by centrifugation (once or twice) at 3500g for 10 minutes, filtered using Miracloth (Calbiochem) and finally forced through 0.45μm syringe driven filters (Millex). The protein extracts were pre-cleared by mixing with 30 μl of washed protein A agarose beads (Millipore) for 20 minutes at 4^o^C and centrifugation at 3500g for 5 minutes. An input aliquot was removed from the supernatant, then GFP-tagged proteins were immunoprecipitated by incubation with 25 μl GFP-trap agarose beads (Chromotek) for 2 hours at 4^o^C with gentle rocking. The beads were harvested by centrifugation for 1 minute at 2000g, washed six times for one to five minutes in IP buffer, and the proteins eluted in 30 μl 2 X Laemmli buffer (Sigma) at 95^o^C for 2 minutes.

For co-IP experiments, the IP and elution was performed as above, however as an independent assay we used a different antibody to GFP (μMACS GFP-tagged protein isolation kit, Miltenyi Biotech Ltd) than that for IP-MS. The input and IP samples were analysed by Western blotting as described above. The mouse polyclonal antibody to MSI1 was a generous gift from Dr Lars Hennig and was described previously [27]. To generate the sheep polyclonal antibody to CLF, an N-terminal portion of CLF (residues 327 – 525) was expressed in *E. coli* as a fusion to the chitin binding domain using the IMPACT system (NEB) according to manufacturer’s instruction. The purified protein was used to immunise sheep four times at monthly intervals (Scottish Antibody Production Unit, Diagnostics Scotland) and the third, final bleed used for serum. To affinity purify the serum, 10 ml was loaded over a 3ml column of the CLF antigen coupled to sulfo-link resin column (Pierce, Thermo Scientific). The anti-CLF antibodies were eluted as described in [28]. The affinity purified antibodies were used at 1:5000 dilution on Western blots.

Mass spectrometry analysis

For MS analysis, proteins were briefly (10 min) run into SDS-PAGE gels. A band of coomassie-stained gel was excised and the proteins where digested using Trypsin as described elsewhere [29]. In brief, proteins were reduced in 10 mM DTT for 30 min at 37°C, alkylated in 55 mM iodoacetamide for 20 min at room temperature in the dark, and digested overnight at 37°C with 12.5 ng/μL Trypsin (Proteomics Grade, Sigma). The digestion media was then acidified to 0.1% of TFA and spun onto StageTips as described in the literature [30]. Peptides were eluted in 20 μL of 80% acetonitrile in 0.1% TFA and were concentrated to 4 μL (Concentrator 5301, Eppendorf AG). The peptides sample was then diluted to 5 μL by 0.1% TFA for LC-MS/MS analysis.

Analysis was performed using a Velos LTQ-Orbitrap mass spectrometer (Thermofisher Scientific) coupled on-line to an Waters Nano AQUITY UPLC (Waters) or (replicates 2 and 3) in a Q-Exactive mass spectrometer (Thermofisher Scientific) coupled on-line to an Ultimate 3000 RSLCnano System (Thermo Fisher Scientific). Injections were performed in an analytical column with a self-assembled particle frit (Ishihama et al. 2002) and C18 material (ReproSil-Pur C18-AQ 3 μm; Dr. Maisch, GmbH) was packed into a spray emitter (75-μm ID, 8-μm opening, 300-mm length; New Objective) using an air-pressure pump (Proxeon Biosystems). Mobile phase A consisted of water, 5% acetonitrile, and 0.5% formic acid; mobile phase B, consisted of acetonitrile and 0.5% formic acid. The gradient used was 95 min. The peptides were loaded onto the column at a flow rate of 0.7uL/min and eluted at a flow rate of 0.3uL/min according to the gradient: 1 to 5% buffer B for 1 min, then to 32% B for 82 min, then to 35%B for 7 min and to 85%B for 5 min. FTMS spectra were recorded at 60,000 resolution and the twenty most intense peaks of the MS scan were selected in the ion trap for MS2, (normal scan, wideband activation, filling 5.0E5 ions for MS scan, 1.0E4 ions for MS2, maximum fill time 100 msec, dynamic exclusion for 60s sec). For the Q-Exactive, FTMS spectra were recorded at normalized collision energy of 25, 70,000 resolution, AGC 1e6 and max filling time of 20 ms. The 10 most intense peaks of MS scan were selected in the ion trap for MS2 (17,500 resolution, AGC 1e6, maximum fill time 60 ms, dynamic exclusion for 60s).

Searches were conducted using Mascot software (version 2.4.1) against a database containing *Arabidopsis thaliana* sequences (UniprotKB_aratha downloaded on 15.03.2015). The search parameters were: MS accuracy, 6 ppm; MS/MS accuracy, 0.6 Da; enzyme, trypsin; allowed number of missed cleavages, 2; fixed modification, carbamidometylation on Cysteine; variable modification, oxidation on Methionine.

Sequence retrieval, phylogenetic and synteny analysis

The ALP1 protein and the deduced protein product of *At3g55350* were used in BLASTP and TBLASTN searches to identify the most similar proteins from diverse land plants. The retrieved proteins were used in reverse BLASTP searches against the *Arabidopsis* genome and in all cases retrieved the original query protein as the best hit. Where genome sequences were available, the sequences were retrieved by interrogating the Genbank and SwissProt databases. In other cases, the transcript sequences were obtained by querying the OneKP (one thousand plant genomes) database (<http://www.onekp.com>). Access to the *Chara braunii* transcriptome was generously provided by the group of Liam Dolan at the University of Oxford (see acknowledgements for full details). The retrieved sequences were aligned using MUSCLE [31] implemented within the MEGA6 package [32] and the alignments manually edited. The phylogenetic trees were produced using the Maximum Likelihood method based on the JTT matrix-based model [33] implemented within MEGA6 [32]. 200 bootstrap replicates were performed. Synteny analysis was performed using the program Synteny Viewer available on the Arabidopsis Information Resource (TAIR). In addition, the syntenic regions were further confirmed manually using TBLASTN to retrieve the most similar proteins to those neighbouring *ALP1* in *Arabidopsis*.

1. Kapitonov VV, Jurka J (2004) Harbinger transposons and an ancient HARBI1 gene derived from a transposase. DNA Cell Biol 23: 311-324.

2. Zhang X, Jiang N, Feschotte C, Wessler SR (2004) PIF- and Pong-like transposable elements: distribution, evolution and relationship with Tourist-like miniature inverted-repeat transposable elements. Genetics 166: 971-986.

3. Kelley LA, Mezulis S, Yates CM, Wass MN, Sternberg MJ (2015) The Phyre2 web portal for protein modeling, prediction and analysis. Nat Protoc 10: 845-858.

4. Lopez-Vernaza M, Yang S, Muller R, Thorpe F, de Leau E, et al. (2012) Antagonistic roles of SEPALLATA3, FT and FLC genes as targets of the polycomb group gene CURLY LEAF. PLoS One 7: e30715.

5. Alvarez-Venegas R, Pien S, Sadder M, Witmer X, Grossniklaus U, et al. (2003) *ATX1*, an *Arabidopsis* Homolog of *Trithorax*, Activates Flower Homeotic Genes. Curr Biol 13: 627-637.

6. Hartwig B, James GV, Konrad K, Schneeberger K, Turck F (2012) Fast isogenic mapping-by-sequencing of ethyl methanesulfonate-induced mutant bulks. Plant Physiol 160: 591-600.

7. Goodrich J, Puangsomlee P, Martin M, Long D, Meyerowitz EM, et al. (1997) A Polycomb-group gene regulates homeotic gene expression in Arabidopsis. Nature 386: 44-51.

8. Bouveret R, Schonrock N, Gruissem W, Hennig L (2006) Regulation of flowering time by Arabidopsis MSI1. Development 133: 1693-1702.

9. Chanvivattana Y, Bishopp A, Schubert D, Stock C, Moon YH, et al. (2004) Interaction of Polycomb-group proteins controlling flowering in Arabidopsis. Development 131: 5263-5276.

10. Soppe WJ, Bentsink L, Koornneef M (1999) The early-flowering mutant efs is involved in the autonomous promotion pathway of Arabidopsis thaliana. Development 126: 4763-4770.

11. Sung ZR, Belachew A, Shunong B, Bertrand-Garcia R (1992) EMF, an *Arabidopsis* Gene Required for Vegetative Shoot Development. Science 258: 1645-1647.

12. Yang C-H, Chen L-J, Sung ZR (1995) Genetic Regulation of Shoot Development in *Arabidopsis*: Role of the EMF Genes. Dev Biol 169: 421-435.

13. Michaels SD, Amasino RM (1999) FLOWERING LOCUS C encodes a novel MADS domain protein that acts as a repressor of flowering. Plant Cell 11: 949-956.

14. Eshed Y, Baum SF, Bowman JL (1999) Distinct mechanisms promote polarity establishment in carpels of Arabidopsis. Cell 99: 199-209.

15. Weigel D, Alvarez J, Smyth DR, Yanofsky MF, Meyerowitz EM (1992) LEAFY controls floral meristem identity in Arabidopsis. Cell 69: 843-859.

16. Gaudin V, Libault M, Pouteau S, Juul T, Zhao G, et al. (2001) Mutations in LIKE HETEROCHROMATIN PROTEIN 1 affect flowering time and plant architecture in Arabidopsis. Development 128: 4847-4858.

17. Kiyosue T, Ohad N, Yadegari R, Hannon M, Dinneny J, et al. (1999) Control of fertilization-independent endosperm development by the MEDEA polycomb gene in Arabidopsis. Proc Natl Acad Sci U S A 96: 4186-4191.

18. Monfared MM, Carles CC, Rossignol P, Pires HR, Fletcher JC (2013) The ULT1 and ULT2 trxG genes play overlapping roles in Arabidopsis development and gene regulation. Mol Plant 6: 1564-1579.

19. Fletcher JC (2001) The *ULTRAPETALA* Gene Controls Shoot and Floral Meristem Size in *Arabidopsis*. Development 128: 1323-1333.

20. Schubert D, Primavesi L, Bishopp A, Roberts G, Doonan J, et al. (2006) Silencing by plant Polycomb-group genes requires dispersed trimethylation of histone H3 at lysine 27. EMBO J 25: 4638-4649.

21. Ossowski S, Schneeberger K, Clark RM, Lanz C, Warthmann N, et al. (2008) Sequencing of natural strains of Arabidopsis thaliana with short reads. Genome Res 18: 2024-2033.

22. Schneeberger K, Hagmann J, Ossowski S, Warthmann N, Gesing S, et al. (2009) Simultaneous alignment of short reads against multiple genomes. Genome Biol 10: R98.

23. Robinson MD, McCarthy DJ, Smyth GK (2010) edgeR: a Bioconductor package for differential expression analysis of digital gene expression data. Bioinformatics 26: 139-140.

24. Nakagawa T, Kurose T, Hino T, Tanaka K, Kawamukai M, et al. (2007) Development of series of gateway binary vectors, pGWBs, for realizing efficient construction of fusion genes for plant transformation. J Biosci Bioeng 104: 34-41.

25. Reimer JJ, Turck F (2010) Genome-wide mapping of protein-DNA interaction by chromatin immunoprecipitation and DNA microarray hybridization (ChIP-chip). Part A: ChIP-chip molecular methods. Methods Mol Biol 631: 139-160.

26. Derkacheva M, Steinbach Y, Wildhaber T, Mozgova I, Mahrez W, et al. (2013) Arabidopsis MSI1 connects LHP1 to PRC2 complexes. EMBO J 32: 2073-2085.

27. Ach RA, Taranto P, Gruissem W (1997) A conserved family of WD-40 proteins binds to the retinoblastoma protein in both plants and animals. Plant Cell 9: 1595-1606.

28. Chen RH, Waters JC, Salmon ED, Murray AW (1996) Association of spindle assembly checkpoint component XMAD2 with unattached kinetochores. Science 274: 242-246.

29. Shevchenko A, Wilm M, Vorm O, Jensen ON, Podtelejnikov AV, et al. (1996) A strategy for identifying gel-separated proteins in sequence databases by MS alone. Biochem Soc Trans 24: 893-896.

30. Rappsilber J, Mann M, Ishihama Y (2007) Protocol for micro-purification, enrichment, pre-fractionation and storage of peptides for proteomics using StageTips. Nat Protoc 2: 1896-1906.

31. Edgar RC (2004) MUSCLE: multiple sequence alignment with high accuracy and high throughput. Nucleic Acids Res 32: 1792-1797.

32. Tamura K, Stecher G, Peterson D, Filipski A, Kumar S (2013) MEGA6: Molecular Evolutionary Genetics Analysis version 6.0. Mol Biol Evol 30: 2725-2729.

33. Jones DT, Taylor WR, Thornton JM (1992) The rapid generation of mutation data matrices from protein sequences. Comput Appl Biosci 8: 275-282.
