# Supplementary figures and images for: Kicking against the PRCs – A Domesticated Transposase Antagonises Silencing Mediated by Polycomb Group Proteins and Is an Accessory Component of Polycomb Repressive Complex 2
Source: PLoS Genet. 2015 Dec 7;11(12):e1005660. doi: 10.1371/journal.pgen.1005660 (PMC4671723; doi:10.1371/journal.pgen.1005660)

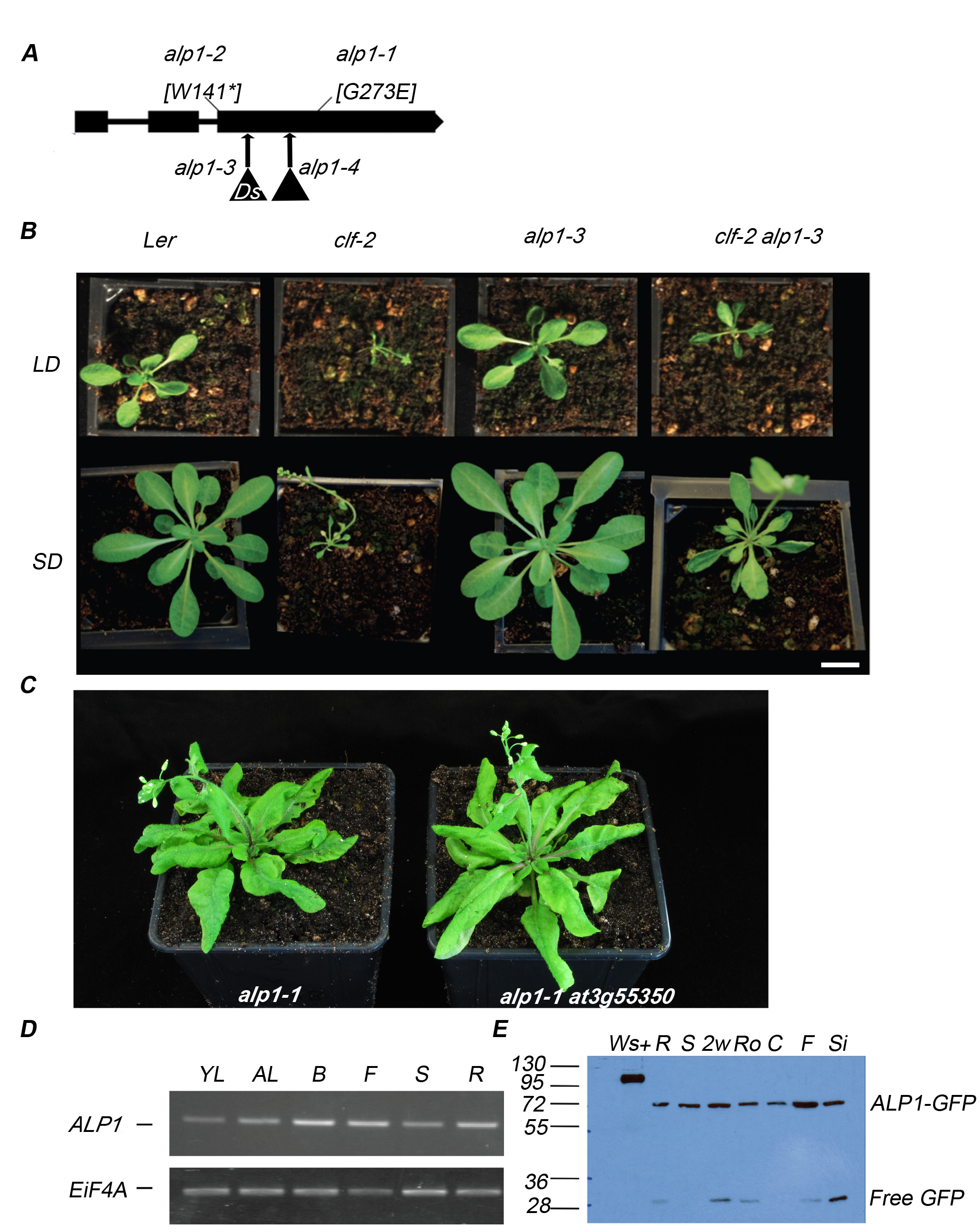

Supplement: S1 Fig — (A) ALP1 gene structure showing the position of the lesions in four independent alleles. Exons are shown as boxes, introns as lines, T-DNA insertion as a triangle. The alp1-3 allele (CSHL ET1398) harbours a modified Ds transposon insertion. Molecular analysis of alp1-4 revealed that the T-DNA insert is complex, containing at least two T-DNA copies in inverted orientation and a 62 bp deletion of ALP1 sequences flanking the insert; however there were no major rearrangements of the ALP1 locus. (B) alp1-3 partially suppresses the null clf-2 mutation, particularly in short days (SD). Plants were 26 days old (LD) or 51 days old (SD). Scale bar 1cm. (C) Double mutant between alp1-1 and at3g55350 (Salk_122829). Plants grown in long days. There was no obvious difference in the rosette, floral phenotype, or flowering time. Plants shown are siblings in progeny of an alp1-1 individual heterozygous for the T DNA insertion allele at at3g55350. (D) RT PCR analysis of ALP1 expression in different tissues. YL, young leaves; AL, adult leaves; B, flower buds; F,flowers; S, seedlings; R, roots. EiF4A is a reference gene used to normalise cDNA amount used in each experiment. (E) Western blot analysis of the presence of ALP1-GFP in tissues. Total crude proteins wereextracted from a variety of tissues including roots (R), inflorescence stems (S), 2-week-old seedlings (2w), rosette leaves (Ro), cauline leaves (C), flower bud and inflorescence (F) and siliques (S) of transgenic pALP1::ALP1-GFP alp1-4 plants, and then analysed by Western blotting using a mouse monoclonal antibody against GFP. Protein extracts from Ws and pLHP1::LHP1-GFP (+) were also included as negative and positive control, respectively. (TIF) [file pgen.1005660.s002.tif]

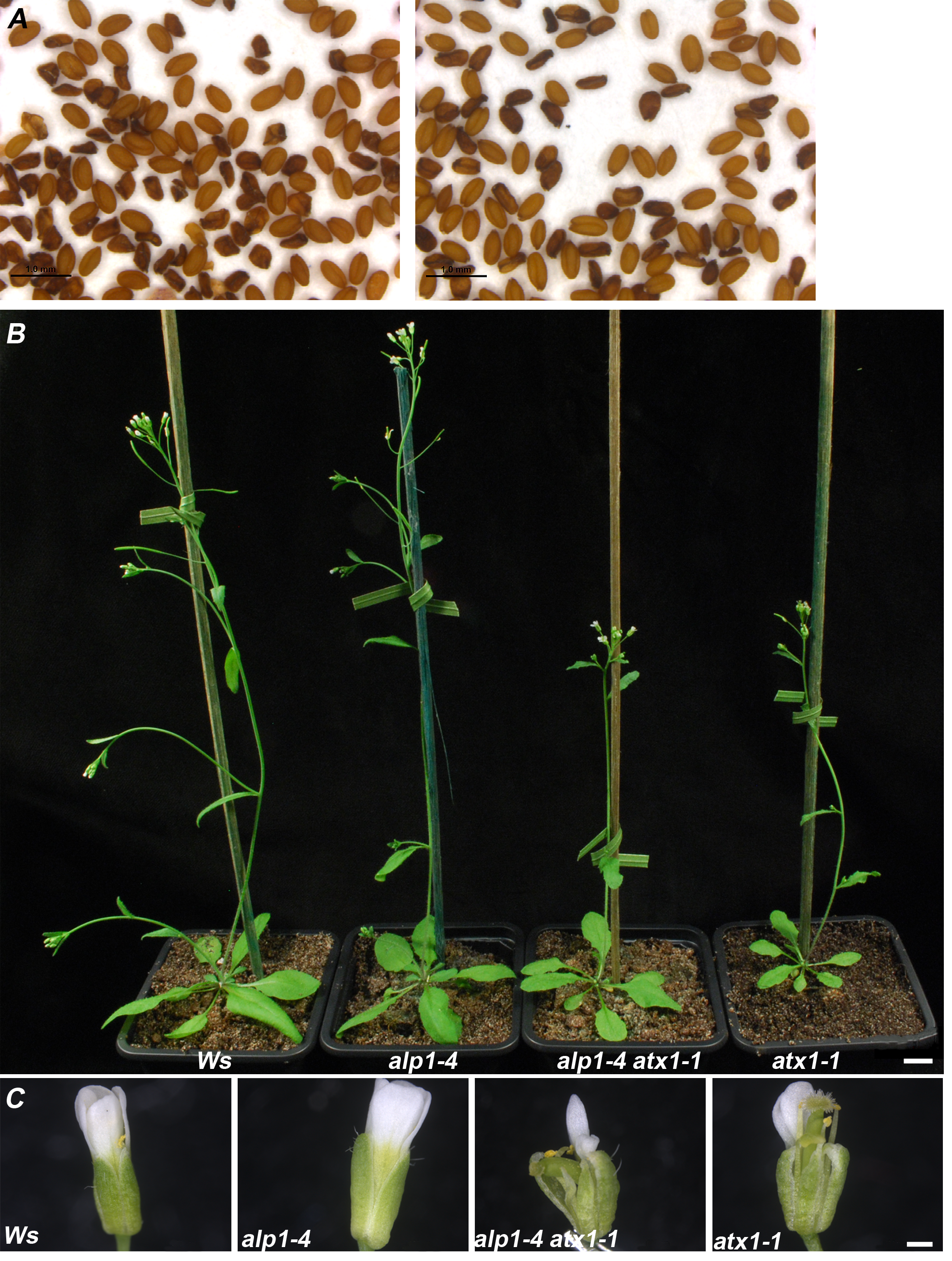

Supplement: S2 Fig — (A) Seed from mea-emb173/+ plants (left panel) and mea-emb173/+ alp1-4 plants (right panel). Both plants segregate lighter coloured plump, seed and darker coloured collapsed seed due to the zygotic lethality of maternally inherited mea-emb173. Scale bar 1mm. (B) Double mutants of alp1-4 atx1-1, long day grown plants. The alp1-4 atx1-1 double mutant does not enhance the mild atx1 phenotype. Scale bar 1 cm. (C) Floral phenotypes. Flowers of the double mutants were similar to those of the atx1-1 single mutant with no obvious enhancement. Scale bar 500 μm. (TIF) [file pgen.1005660.s003.tif]

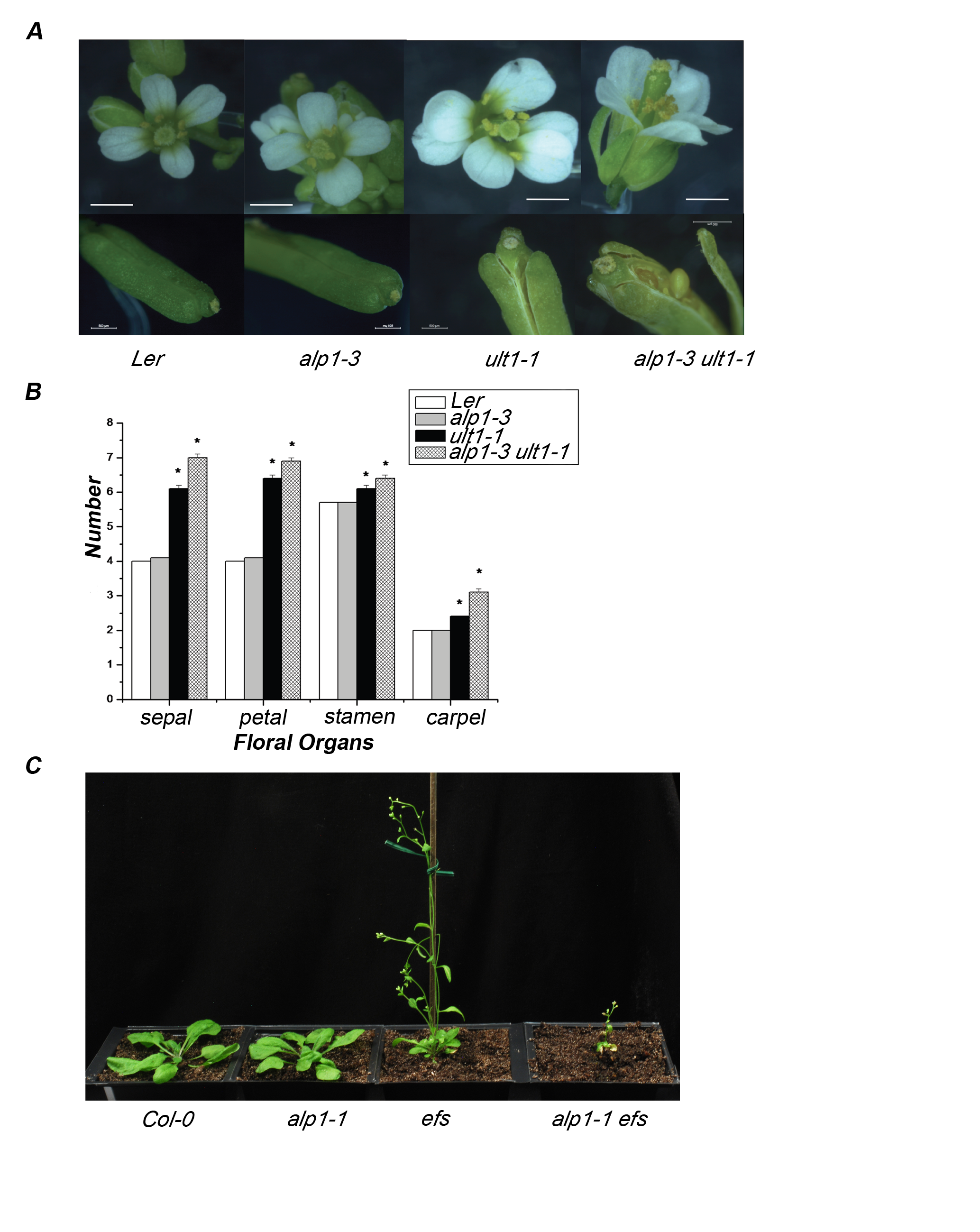

Supplement: S3 Fig — (A) The flowers and siliques of alp1-3 and ult1-1 mutants. The silique of alp1-3 ult1-1 was composed of four carpels, while in ult1-1, it was usually three. The ult1-1 and alp1-3 ult1-1 flowers typically had extra petals relative to wild-type. Scale bars, upper panel, 1 mm; lower panel, 0.5 mm. (B) Statistical analysis of floral organ numbers in alp1-3 and ult1-1 mutants. The floral organs of the initial 10 flowers on primary inflorescence stems were counted and the average numbers of each floral organ are shown with 1 standard error of the mean as error bars. Data were collected from 11–19 individual plants. The stars mark the data that are significantly different from data of wild-type plants in one way ANOVA tests (p<0.001). Note that there was also a significant difference between alp1-3 ult1-1 and ult1-1 (p<0.001). (C) Double mutants between alp1-1 and efs (Salk_026442, also known as sdg8-2) in uniform Col-0 background. The double mutants were much smaller and more dwarved than the single mutants. (TIF) [file pgen.1005660.s004.tif]

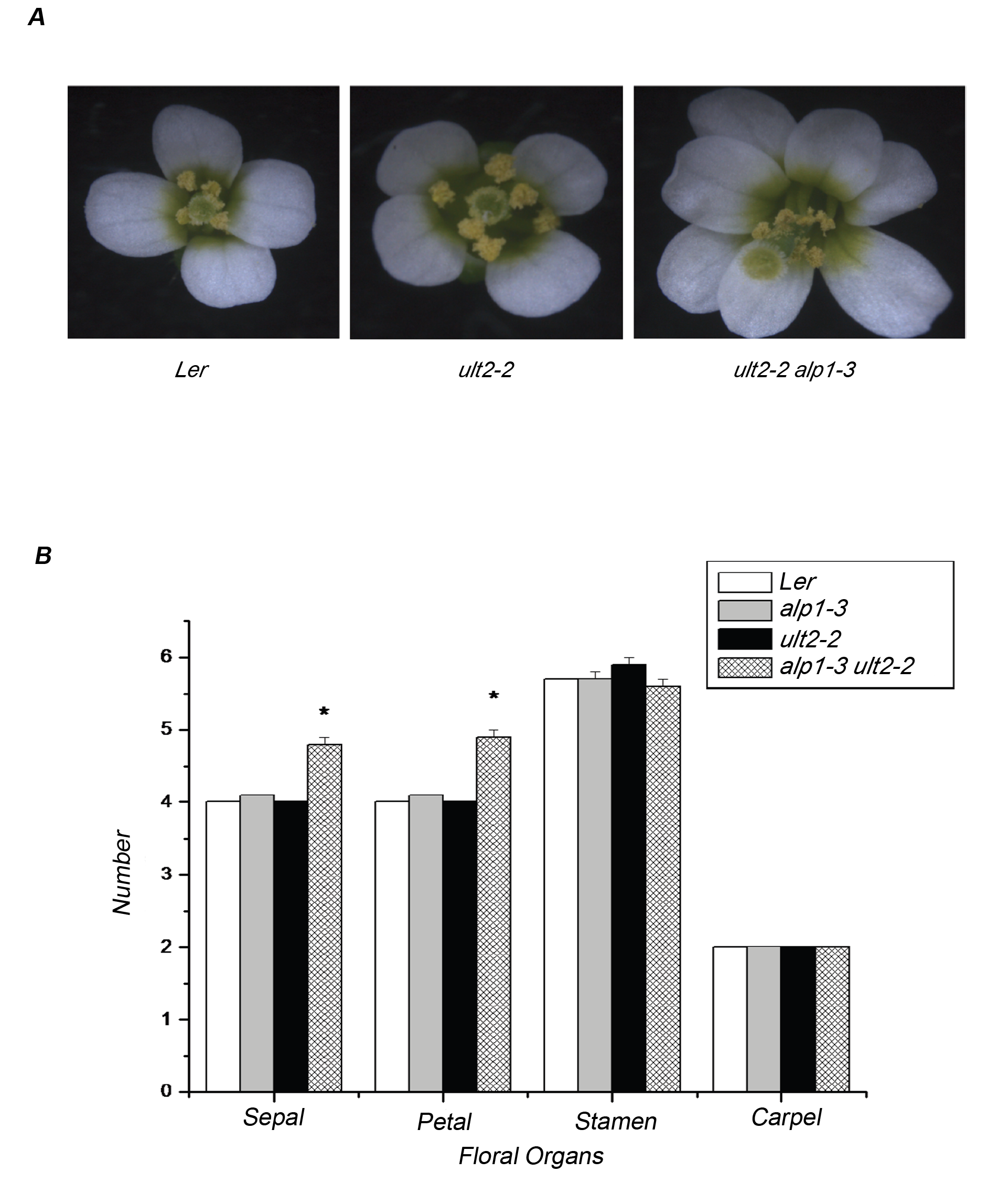

Supplement: S4 Fig — (A) The flower of alp1-3 and ult2-2 mutants. In ult2-2 and alp1-3, the numbers of floral organs are normal, whereas the double mutant alp1-3 ult2-2 displayed extra petals. Photographs were taken under the same scale. (B) Statistical analysis of floral organ numbers in alp1-3 and ult2-2 mutants. The floral organs of initial 10 flowers on primary inflorescence stems were counted and the average numbers of each floral organ are shown with 1 standard error of the mean as error bars. Data were collected from 11–19 individual plants. The stars mark the data that are significantly different compared with data of wild type plants (p<0.001, ANOVA test). (TIF) [file pgen.1005660.s005.tif]

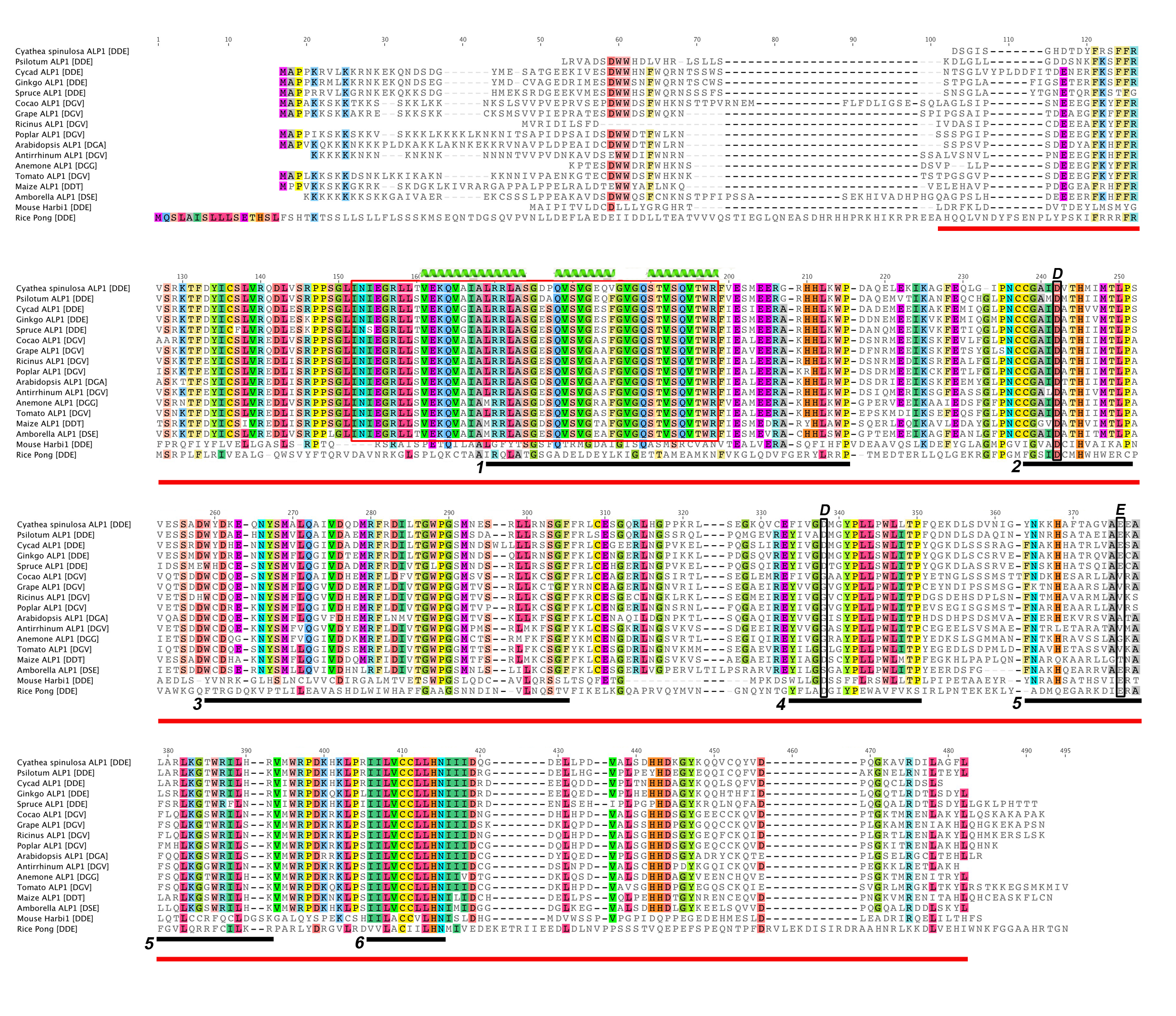

Supplement: S5 Fig — Alignment between selected land plant ALP1 proteins, rice Pong transposase and mouse Harbi1 nuclease made using MUSCLE. Amino acids are shaded according to the RasMol colour scheme based on their properties. Black lines underneath the alignment indicate six regions previously found to be conserved between PIF/Harbinger nucleases [1], the red line a large region of conservation between plant PONG transposases [2]. The black boxes indicate the position of the DDE catalytic triad that is conserved amongst tranposases. Analysis of the Arabidopsis ALP1 protein sequence using the structural prediction program PHYRE [3] identified a potential helix turn helix turn helix motif with low similarity to the DNA binding domain of homeodomain class proteins. The position of the helices is indicated in green above the alignment. The sequence identities are as described in the legend to Fig 2, mouse Harbi1 is Genbank GI:154759331. (TIF) [file pgen.1005660.s006.tif]

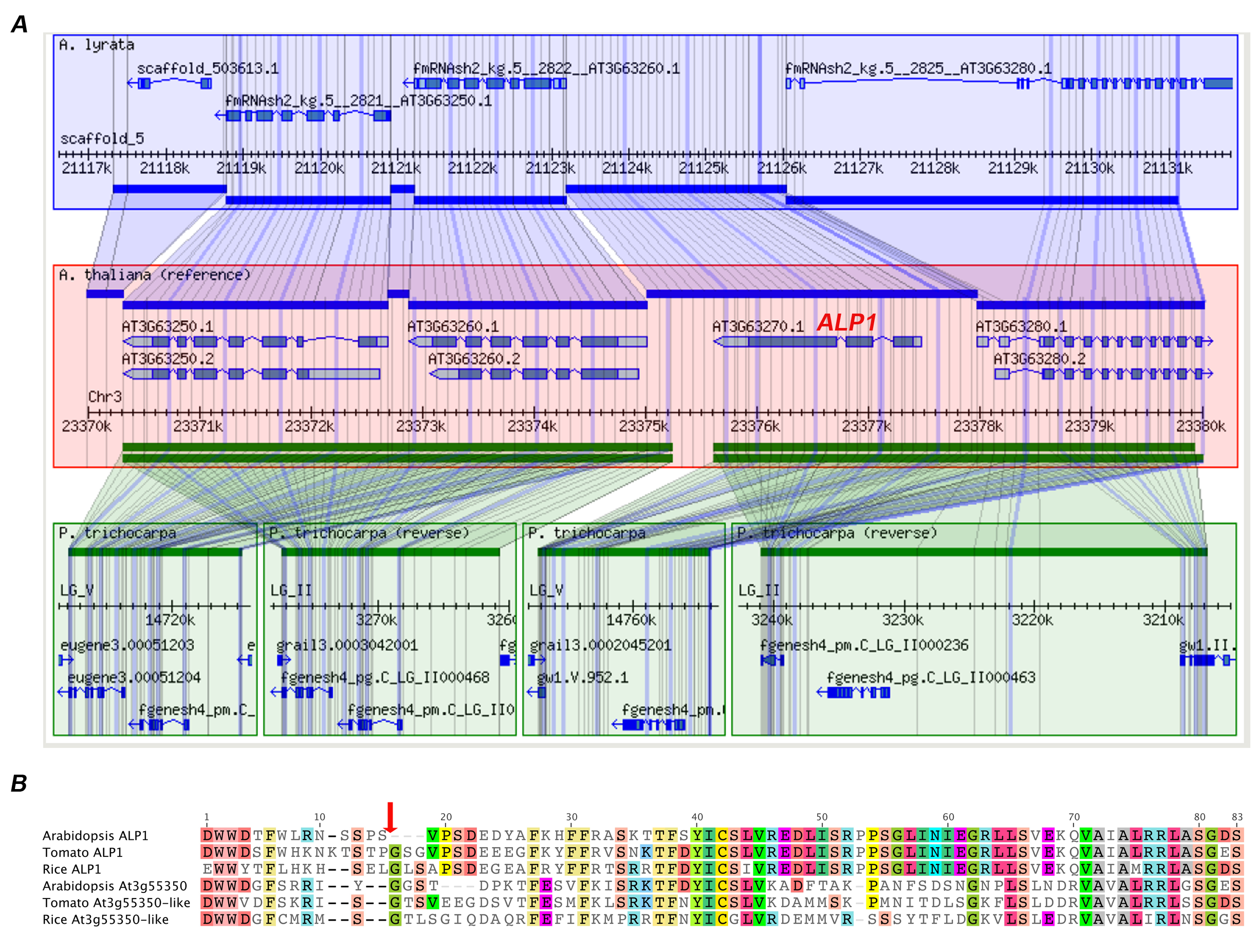

Supplement: S6 Fig — (A) Comparison of the genomic region around ALP1 in Arabidopsis thaliana with corresponding regions in Arabidopsis lyrata and Populus trichocarpa. The genes neighbouring ALP1, their orientation and relative order are conserved between the three species, indicating that ALP1 has not transposed at least in the time since these species diverged from their common ancestor. Futher manual inspection confirmed that the genes neighbouring Populus trichocarpa ALP1 on LGII retrieve the genes neighbouring ALP1 in Arabidopsis as best hits in reciprocal TBLASTN searches. (B) Intron position is conserved between ALP1 and At3g55350 genes in various angiosperm species. The red arrow indicates the position at which the intron interrupts the predicted protein sequences of the different genes. The alignment of a portion of the protein sequences indicates that the intron is at the same position in all genes, strongly suggesting a common evolutionary origin for ALP1 and At3g55350. With the exception of Arabidopsis ALP1 which contains two introns, all the other genes contain a single intron. (TIF) [file pgen.1005660.s007.tif]
