# Supplementary material for: Kicking against the PRCs – A Domesticated Transposase Antagonises Silencing Mediated by Polycomb Group Proteins and Is an Accessory Component of Polycomb Repressive Complex 2
Source: PLoS Genet. 2015 Dec 7;11(12):e1005660. doi: 10.1371/journal.pgen.1005660 (PMC4671723; doi:10.1371/journal.pgen.1005660)
Supplement: S5 Table — Origin of the different genetic materials used including nature of the mutation and the genetic background in which the mutant was isolated. (DOCX) [file pgen.1005660.s012.docx]

| Name | Locus | Mutation type | Background | Source or reference |
| --- | --- | --- | --- | --- |
| *atx1-1* | *AT2G31650* | T-DNA insertion | Ws | Z. Avramova [5] |
| *atx2-2* | *AT1G05830* | T-DNA insertion  (SALK_117262) | Col | ABRC |
| *alp1-1* | *AT3G63270* | Missense | Col | [6] |
| *alp1-3* | *AT3G63270* | Enhancer trap line (CSHL_ET1398) | L*er* | NASC |
| *alp1-4* | *AT3G63270* | T-DNA insertion | Ws | [4] |
| *clf-2* | *AT2G23380* | *Ds* insertion | L*er* | [7] |
| *clf-29* | *AT2G23380* | T-DNA insertion  Salk_021003 | Col | [8] |
| *clf-50* | *AT2G23380* | Deletion | Ws | [9] |
| *efs* | *AT1G77300* | Missense | L*er* | [10] |
| *emf1-1* | *AT5G11530* | Nonsense | Col | R. Sung [11] |
| *emf2-3* | *AT5G51230* | 35bp deletion | Col | R. Sung [12] |
| *flc-3* | *AT5G10140* | 104 bp deletion | Ws (BC2 from Col-0) | R. Amasino [13] |
| *gym-5* | *AT2G25170* | Missense | L*er* | J. Bowman [14] |
| *lfy-5* | *AT5G61850* | Missense | L*er* | NASC [15] |
| *lhp1-2* | *AT5G17690* | Nonsense | Ws | V. Gaudin [16] |
| *mea-emb173* | *AT1G02580* | Nonsense | Ws | M. Luo [17] |
| *swn-3* | *AT4G02020* | T-DNA insertion  (Salk_050195) | Col | [9] |
| *ult2-2* | *AT2G20825* | Nonsense | L*er* | C. Carles [18] |
| *ult1-1* | *AT4G28190* | Missense | L*er* | C. Carles [19] |
| *35S::GFP-CLF* | Transgene | *clf-50* | Ws | [20] |
| *35S::GFP* | Transgene | - | Ws | [20] |
